# A Fish MDA5 Integrates Canonical RIG‐I and MDA5 Ligand Recognition

**DOI:** 10.1002/advs.78318

**Published:** 2026-10-14

**Authors:** Wenxing Li, Yuan Feng, Huazhi Chen, Jiamei Liu, Álvaro Fernández Montero, Jiangtao Gao, Jingqun Ao, Xinhua Chen

**Affiliations:** ^1^ State Key Laboratory of Mariculture Breeding Key Laboratory of Marine Biotechnology of Fujian Province College of Marine Sciences Fujian Agriculture and Forestry University Fuzhou China; ^2^ Grupo de investigación en acuicultura (GIA) IU‐ECOAQUA Universidad De Las Palmas De Gran Canaria Las Palmas Spain; ^3^ College of Biomedical Sciences Fujian Agriculture and Forestry University Fuzhou China

**Keywords:** antiviral signaling, innate immunity, large yellow croaker (*Larimichthys crocea*), MDA5, RIG‐I, RNA sensing

## Abstract

RIG‐I and MDA5 are cytosolic RNA sensors that recognize distinct viral ligands in mammals, yet RIG‐I has been independently lost from multiple teleost lineages. How RIG‐I‐deficient vertebrates preserve recognition of RIG‐I‐associated viral RNA remains poorly understood. Here, we show that MDA5 from the large yellow croaker (*Larimichthys crocea*, *Lc*), a teleost species lacking RIG‐I, recognizes both 5′‐triphosphate dsRNA (5′ppp‐dsRNA), the canonical ligand of RIG‐I, and long dsRNA, the classical ligand of MDA5. Its C‐terminal domain (CTD) is essential for the recognition of both ligands, with residues specifically required for sensing the 5′ppp moiety. Structural modeling, molecular dynamics simulations, and mutational validation support a model in which a flexible RNA‐capping loop in the CTD confers *Lc*MDA5 a conformational plasticity, allowing the CTD to cap the 5′ppp‐dsRNA terminus for RIG‐I‐like recognition or orienting the CTD parallel to long‐dsRNA axis for canonical MDA5 recognition. Disruption of 5′ppp recognition impaired *Lc*MDA5‐mediated antiviral activity against a RIG‐I‐sensitive virus. Downstream, *Lc*MDA5 signals through an integrated MAVS–STING–TBK1/IRF3 axis to drive type I IFN responses to both RNA ligands. Our findings reveal an unexpectedly flexible architecture of vertebrate cytosolic RNA sensing in which a single RLR combines ligand‐recognition and signaling properties classically partitioned between RIG‐I and MDA5.

## Graphical abstract

These results show that the large yellow croaker lacks RIG‐I, yet its MDA5 senses both 5′ppp‐dsRNA and long dsRNA. A flexible RNA‐capping loop in the MDA5 C‐terminal domain enables two ligand‐binding modes. This dual recognition signals through MAVS–STING–TBK1/IRF3 and induces type I interferons, revealing unexpected plasticity in vertebrate antiviral RNA sensing.

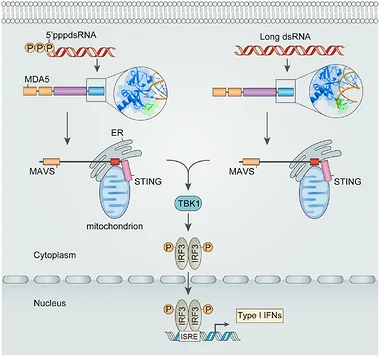

## 1 Introduction

RIG‐I‐like receptors (RLRs) are cytosolic pattern recognition receptors (PRRs) that are essential for detecting double‐stranded RNA (dsRNA), a common pathogen‐associated molecular pattern (PAMP) produced as a replication intermediate of viruses [1, 2]. Retinoic acid‐inducible gene I (RIG‐I) is known to detect short dsRNA bearing a 5′‐triphosphate (5′ppp) group, while melanoma differentiation‐associated gene 5 (MDA5) recognizes long, blunt‐ended dsRNA [3, 4]. Upon recognition of dsRNA ligands, RIG‐I and MDA5 interact with the mitochondrial antiviral signaling protein (MAVS), which is localized on the mitochondrial outer membrane, to trigger type I interferon (IFN) production [5]. In mammals, the stimulator of interferon genes protein (STING) selectively enhances RIG‐I‐mediated signaling but plays a limited role in MDA5‐driven responses [6]. STING binds MAVS at mitochondria‐associated endoplasmic reticulum membranes and promotes the activation of TANK‐binding kinase 1 (TBK1), leading to interferon regulatory factor 3 (IRF3) phosphorylation and type I IFN production [7, 8].

Teleosts possess a well‐developed type I IFN system, in which RIG‐I and MDA5 act as primary cytosolic inducers [9]. Teleost RIG‐I and MDA5 maintain domain architectures similar to their mammalian homologs [10], and both signal through MAVS [11, 12]. Increasing evidence suggests that teleost STING participates in RLR‐mediated antiviral signaling. In crucian carp, expression of a dominant‐negative STING mutant abolishes type I IFN activation mediated by both RIG‐I and MDA5 [13], suggesting that STING plays an essential role in RLR‐mediated signaling in fish [13, 14]. In epithelioma papulosum cyprini (EPC) cells, overexpression of the isolated C‐terminal region of zebrafish STING significantly suppressed RIG‐I‐ and MAVS‐mediated type I IFN production [15]. However, whether fish STING interacts directly with RLRs or MAVS, and how it shapes the RLR signaling cascade, have not been determined.

Despite the pivotal role of RLRs in antiviral immunity, RIG‐I is absent from many teleost lineages, including Acanthomorpha, Clupeiformes, and Polypteriformes, which together represent a large proportion of modern bony fishes [16, 17, 18]. More generally, the evolutionary persistence or loss of genes can be influenced by their functional dispensability, which is shaped by the presence of functional redundancy or alternative pathways [19]. These buffering mechanisms can reduce the fitness consequences associated with gene loss [19]. The widespread absence of RIG‐I in teleosts provides an opportunity to investigate how antiviral RNA sensing can be maintained when a canonical receptor is absent. In the RIG‐I‐deficient sciaenid fish miiuy croaker (*Miichthys miiuy*), MDA5 has been shown to directly recognize viral 5′ppp‐RNA by its C‐terminal domain, activate type I IFN production, and restrict viral replication [18]. However, the molecular mechanisms underlying RNA recognition by fish MDA5 remain unresolved. Clarifying these mechanisms is critical to understanding how robust antiviral sensing can be maintained despite the loss of RIG‐I.

Large yellow croaker (*Larimichthys crocea*), a commercially important sciaenid mariculture species, lacks RIG‐I [20]. Here, we demonstrate that *Lc*MDA5 exhibits dual‐ligand recognition capability. Structural modeling, molecular dynamics simulations, and mutational validation support a model in which a flexible RNA‐capping loop in the CTD endows *Lc*MDA5 with the conformational plasticity, enabling the CTD to cap the 5′ppp‐dsRNA terminus for RIG‐I‐like recognition or orient parallel to the long‐dsRNA axis for canonical MDA5 recognition. Functionally, 5′ppp recognition is critical for *Lc*MDA5 antiviral activity against a RIG‐I‐sensitive virus. Further studies reveal that *Lc*MDA5 employs both MAVS and STING to activate the TBK1/IRF3–IFN pathway. Hierarchically, *Lc*STING functions downstream of *Lc*MAVS in the *Lc*MDA5‐mediated signaling cascade. These findings suggest that dsRNA sensors may exhibit greater functional plasticity than previously appreciated and highlight the mechanistic complexity of dsRNA recognition.

## 2 Results

### 2.1 The *L. crocea* Genome Does Not Contain any Sequence Remnants of RIG‐I

We previously reported the absence of RIG‐I in the *L. crocea* genome [20]. To exclude potential technical artifacts, several complementary analyses were performed. First, we examined the conserved *topors*‐*Aco1* syntenic block (Figure 1A), which contains the RIG‐I locus in other vertebrates. *De novo* gene prediction analysis of the corresponding contig in the *L. crocea* genome (GCA_000972845.2, chr XIV: 6176481–13709732) confirmed the absence of RIG‐I orthologs. Second, we conducted a tBLASTn search against the entire *L. crocea* genome using the zebrafish RIG‐I protein sequence as the query. All candidate hits were verified by BLASTp, but no RIG‐I homolog was identified. Third, we designed degenerate primer pairs targeting conserved regions of fish RIG‐I (Figure 1B), ensuring that the amplified region was not present in the laboratory of genetics and physiology 2 (LGP2) or MDA5 genes. Two primer pairs (Table S1) successfully amplified the target sequence from zebrafish genomic DNA, but no corresponding product was obtained from *L. crocea* (Figure 1C). These results confirm the genuine absence of RIG‐I. Functionally linked genes are often co‐eliminated during evolution [19]. Therefore, we examined the status of RING finger protein 135 (RNF135), the most important RIG‐I regulator, which activates RIG‐I by ubiquitinating its C‐terminal region [21]. The *L. crocea* genome analysis and synteny comparison confirmed the absence of RNF135 from its expected genomic locus (Figure 1D). These findings indicate that the RIG‐I‐dependent cytosolic dsRNA‐sensing pathway is absent in *L. crocea* and is likely supplanted by alternative mechanisms. To test this hypothesis, we transfected large yellow croaker head kidney (LYCK) cells with 5′ppp‐dsRNA using liposome‐based transfection, a method that delivers dsRNA to the cytoplasm [22, 23]. Transcript levels of type I IFNs (IFNi, IFNd, and IFNh) and interferon‐stimulated genes (ISGs; Viperin, PKR, and MxA) were measured at various time points. All tested genes showed significant upregulation (Figure 1E). In contrast, the 5′ppp‐deficient control RNA, which shares the identical sequence with 5′ppp‐dsRNA but lacks the 5′ppp moiety, failed to induce transcription of type I IFNs (Figure S1). These results suggest the presence of an alternative sensor in *L. crocea* that contributes to RLR signaling.

**FIGURE 1:**
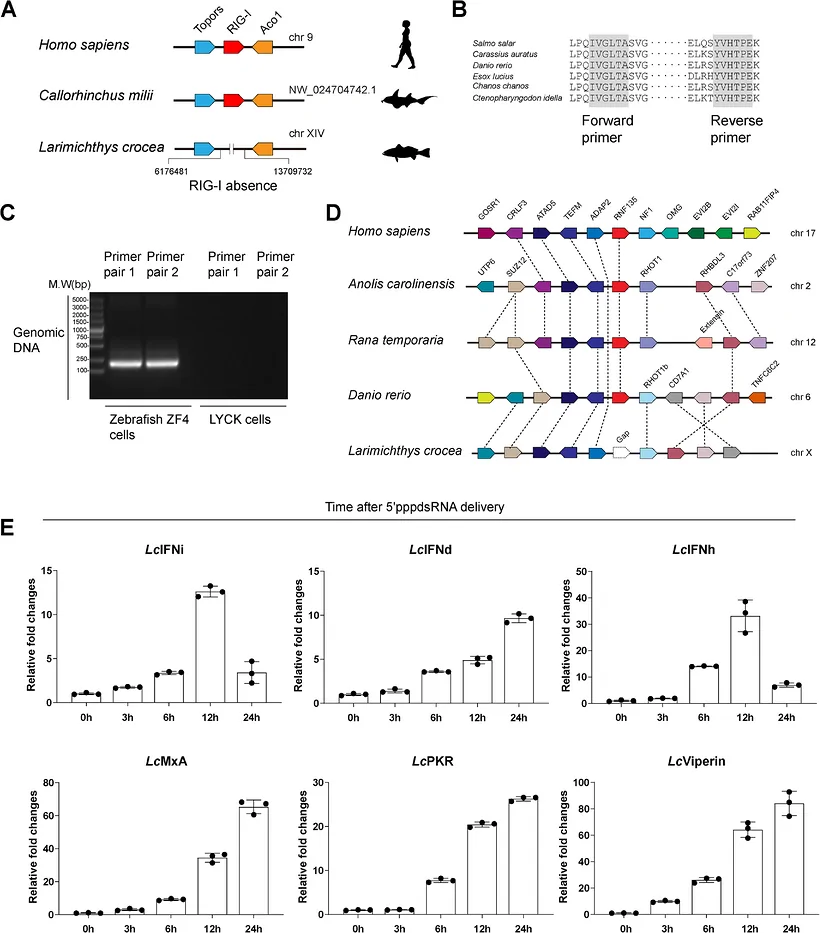
Absence of RIG‐I and its regulator RNF135 in the large yellow croaker. (A) Genomic loci of RIG‐I in human and elephant shark, showing conserved synteny. (B) Design of degenerate PCR primers based on conserved amino acid regions of teleosts RIG‐I. Alignment of RIG‐I amino acid sequences illustrating the region used for primer design. Accession numbers for the aligned sequences are as follows: XP_045569699.1 (*Salmo salar*); XP_026111599.1 (*Carassius auratus*); NP_001293024.1 (*Danio rerio*); XP_028973602.2 (*Esox lucius*); XP_030629349.1 (*Chanos chanos*); XP_051736996.1 (*Ctenopharyngodon Idella* ). (C) PCR‐based detection of the RIG‐I gene in ZF4 and LYCK cell lines. Two primer pairs were used to amplify *RIG‐I* segments from genomic DNA extracted from either ZF4 or LYCK cells. (D) Syntenic analysis of genomic regions surrounding RNF135 in various species. Each polygon represents a gene, and connecting lines indicate conserved genomic blocks. Arrowheads denote transcriptional orientation, and syntenic genes are color‐coded. (E) Induction of type I IFN production by 5′ppp‐dsRNA in LYCK cells. LYCK cells were seeded in 6‐well plates (1×10^6^ cells/well) and transfected with 2.5 µg/mL 5′ppp‐dsRNA. Cells were harvested at the indicated time points for RT‐qPCR analysis of type I IFNs and ISGs. Relative mRNA levels were normalized to β‐actin and are presented as fold induction relative to mock‐transfected control cells. Data are presented as mean ± SD from three independent experiments.

### 2.2 Cytoplasmic 5′ppp‐dsRNA and Long dsRNA Induce Type I IFN Production Through *Lc*MDA5

Foreign dsRNAs are recognized by RLRs and toll‐like receptors (TLRs) [24]. Intracellular delivery of dsRNA via transfection activates cytosolic sensors, including RIG‐I and MDA5 [22, 23]. Extracellular dsRNA can be internalized via endocytosis and detected by endosomal TLR3 [25], as well as by TLR7 specifically in teleosts [26] (Figure 2A). To assess the contribution of *Lc*MDA5 to the sensing of cytoplasmic dsRNA, we employed a genetic knockdown approach. RNA interference experiments were independently performed in LYCK cells targeting *Lc*MDA5, *Lc*TLR3, and *Lc*TLR7 (Figure 2B). This allowed us to clarify the contributions of cytosolic and endosomal sensing pathways. We first assessed the effect of knocking down these dsRNA sensors on the cellular response to intracellular 5′ppp‐dsRNA. Knockdown of *Lc*MDA5, but not *Lc*TLR3 or *Lc*TLR7, reduced the expression of type I IFNs and ISGs following 5′ppp‐dsRNA stimulation (Figure 2C, D), indicating that the type I IFN response is driven primarily by cytosolic sensing of intracellular 5′ppp‐dsRNA, with negligible contribution from endosomal TLRs. A similar situation was observed in cells transfected with high molecular weight poly(I:C) [poly(I:C)‐HMW; 1.5–8 kb] (Figure 2E, F), the classic long dsRNA ligand for MDA5 [27, 28]. To confirm the specificity of *Lc*MDA5 in cytosolic dsRNA sensing, we examined cytokine induction in response to extracellular dsRNA ligands. Knockdown of *Lc*TLR3 or *Lc*TLR7 reduced extracellular poly(I:C)‐HMW‐induced expression of type I IFNs, whereas *Lc*MDA5 knockdown had no effect (Figure 2G). Additionally, *Lc*TLR7 knockdown reduced proinflammatory cytokines (*Lc*TNFα1, *Lc*IL‐6, and *Lc*IFNγ) in response to the ssRNA analog R848, whereas knockdown of *Lc*TLR3 or *Lc*MDA5 had no effect on the up‐regulation of proinflammatory cytokines by ssRNA analog (Figure 2H). These findings show that *Lc*MDA5 is essential for type I IFN induction by intracellular 5′ppp‐dsRNA and long dsRNA. We then examined the phosphorylation status of *Lc*TBK1 and *Lc*IRF3, two downstream signaling molecules of *Lc*MDA5. Knockdown of *Lc*MDA5 reduced phosphorylation of both *Lc*TBK1 and *Lc*IRF3 following transfection with 5′ppp‐dsRNA or poly(I:C)‐HMW (Figure 2I, J), confirming its central role in mediating type I IFN production triggered by cytosolic 5′ppp‐dsRNA and long dsRNA.

**FIGURE 2:**
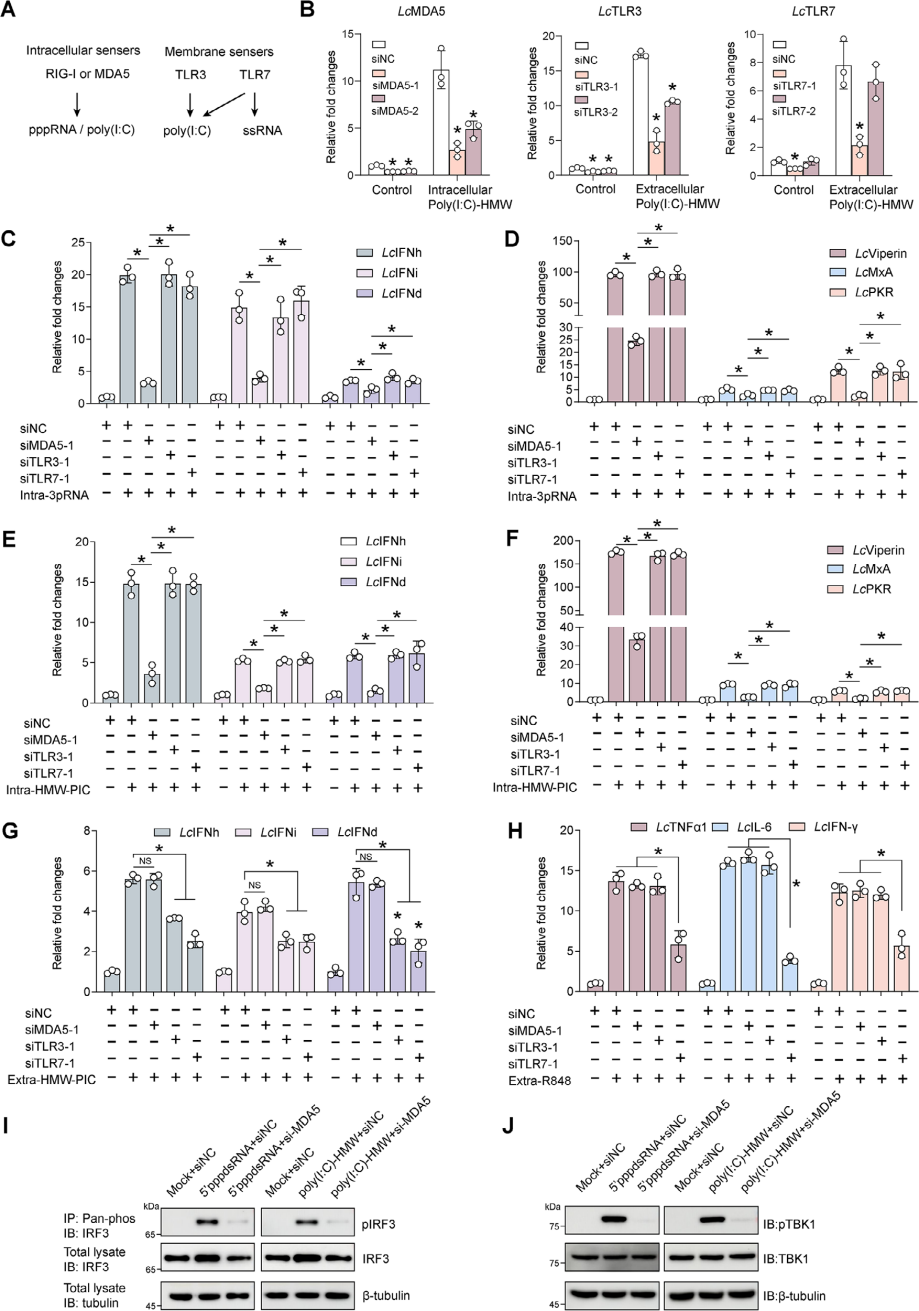
*Lc*MDA5 is essential for cytoplasmic 5′ppp‐dsRNA‐ and poly(I:C)‐HMW‐induced type I IFN production. (A) Illustration of RLR‐ and TLR‐mediated nucleic acid sensing pathways in vertebrates. (B) Knockdown of endogenous *Lc*MDA5, *Lc*TLR3, and *Lc*TLR7 in LYCK cells was achieved using gene‐specific siRNAs. At 24 h post‐siRNA transfection, poly(I:C)‐HMW (2.5 µg/mL) was delivered intracellularly to *Lc*MDA5‐knockdown cells, whereas poly(I:C)‐HMW (10 µg/mL) was added extracellularly to the culture medium of *Lc*TLR3‐ and *Lc*TLR7‐knockdown cells. The knockdown efficiency of all three sensors was confirmed by RT‐qPCR. (C, D) Effect of *Lc*MDA5, *Lc*TLR3, or *Lc*TLR7 knockdown on type I IFN (C) and ISG (D) transcription induced by intracellular 5′ppp‐dsRNA (2.5 µg/mL). (E, F) Effect of *Lc*MDA5, *Lc*TLR3, or *Lc*TLR7 knockdown on type I IFNs (E) and ISGs (F) transcription induced by intracellular poly(I:C)‐HMW (2.5 µg/mL). (G) Effect of *Lc*MDA5, *Lc*TLR3, or *Lc*TLR7 knockdown on the transcription of type I IFNs induced by extracellular poly(I:C)‐HMW (10 µg/mL). (H) Effect of *Lc*MDA5, *Lc*TLR3, or *Lc*TLR7 knockdown on cytokine transcription induced by extracellular R848 (2.5 µg/mL). (I, J) LYCK cells were treated with *Lc*MDA5‐specific siRNA (100 nM) for 24 h, then stimulated with 5′ppp‐dsRNA (2.5 µg/mL) or poly(I:C)‐HMW (2.5 µg/mL) for an additional 24 h. Phosphorylation of *Lc*IRF3 and *Lc*TBK1 was then assessed using a pan‐phosphoserine/threonine antibody and a phospho‐specific TBK1 antibody, respectively. Data are shown as mean ± SD from three independent experiments. (*p < 0.05, two‐tailed unpaired Student's *t*‐test).

### 2.3 *Lc*MDA5 Binds to Both 5′ppp‐dsRNA and Long dsRNA

Given that *Lc*MDA5 is required for cellular responses to two distinct dsRNA ligands (Figure 2), we next examined whether *Lc*MDA5 binds to each ligand. Lysates from LYCK cells expressing Flag‐tagged *Lc*MDA5 were incubated with biotin‐labeled RNAs, and the resulting RNA‐protein complexes were captured via streptavidin pull‐down assays. The results showed that *Lc*MDA5 successfully bound to biotin‐labeled 5′ppp‐dsRNA or biotin‐labeled poly(I:C)‐HMW (Figure 3A, B). The specificity of these interactions was further evaluated using unlabeled 5′ppp‐dsRNA or poly(I:C)‐HMW as competitors, which effectively reduced the pull‐down efficiency of *Lc*MDA5. To validate these findings under physiological conditions, we examined the binding capacity of endogenous *Lc*MDA5 to both 5′ppp‐dsRNA and poly(I:C)‐HMW. We found that endogenous *Lc*MDA5 was efficiently captured by both biotin‐labeled 5′ppp‐dsRNA and poly(I:C)‐HMW (Figure 3C). Crucially, the control RNA that has the same sequence as 5′ppp‐dsRNA but lacks the 5′ppp moiety failed to capture *Lc*MDA5 (Figure 3D), confirming that endogenous recognition of 5′ppp‐dsRNA is dependent on the 5′ppp moiety. Competitive binding assays showed that the interaction between endogenous *Lc*MDA5 and biotin‐labeled 5′ppp‐dsRNA was effectively disrupted by unlabeled 5′ppp‐dsRNA or poly(I:C)‐HMW, whereas the control RNA exerted no competitive effect (Figure 3E). Consistently, the binding of endogenous *Lc*MDA5 to biotin‐labeled poly(I:C)‐HMW was disrupted by unlabeled 5′ppp‐dsRNA or poly(I:C)‐HMW, but not by the control RNA (Figure 3F). Collectively, these results demonstrate that *Lc*MDA5 binds to both 5′ppp‐dsRNA and long‐form dsRNA.

**FIGURE 3:**
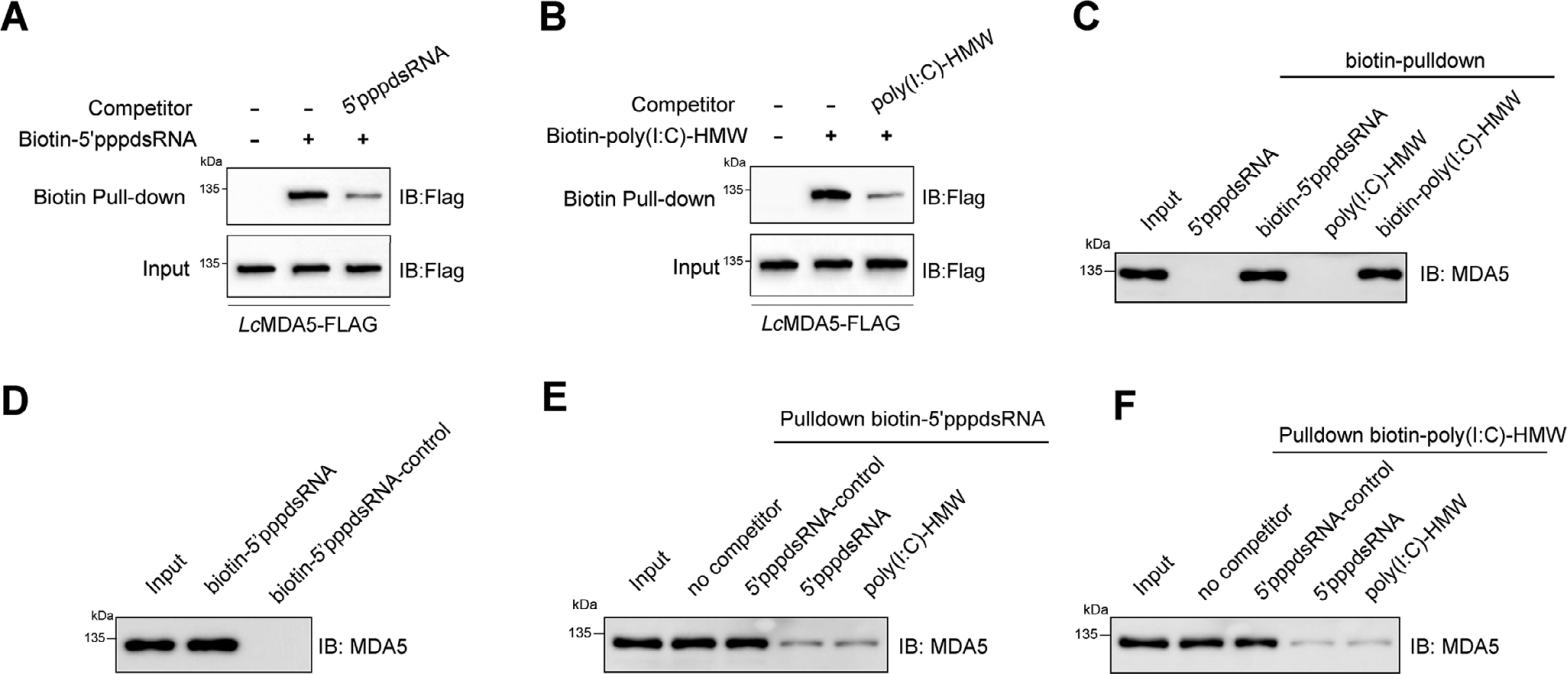
*Lc*MDA5 recognizes both 5′ppp‐dsRNA and long‐form dsRNA. (A, B) RNA pull‐down assays verifying the interaction of ectopically expressed *Lc*MDA5 with biotin‐labeled 5′ppp‐dsRNA (A) or poly(I:C)‐HMW (B). LYCK cells were transfected with a plasmid encoding Flag‐tagged *Lc*MDA5. The cell lysates were incubated with biotinylated 5′ppp‐dsRNA or poly(I:C)‐HMW. Binding specificity was assessed using non‐biotinylated 5′ppp‐dsRNA or poly(I:C) ‐HMW as competitors. (C) Binding analysis of endogenous *Lc*MDA5 to biotin‐labeled 5′ppp‐dsRNA or poly(I:C)‐HMW. (D) The 5′ppp‐deficient control RNA sharing the same sequence as 5′ppp‐dsRNA fails to capture native *Lc*MDA5. (E, F) Competitive binding assays demonstrating the specificity of endogenous *Lc*MDA5 interactions toward 5′ppp‐dsRNA (E) and poly(I:C)‐HMW (F) in the presence of the indicated unlabeled competitors.

### 2.4 Structural Modeling of *Lc*MDA5 to Recognize Both 5′ppp‐RNA Duplex and Long dsRNA

Structural analyses have resolved mammalian RIG‐I bound to a hairpin 5′ppp RNA duplex (PDB: 4AY2) and MDA5 bound to a 15‐mer blunt‐ended dsRNA representing a minimal segment of long dsRNA (PDB: 7BKQ) [29, 30]. Because RLRs recognize foreign RNA in a sequence‐independent manner [31, 32], we reasoned that if *Lc*MDA5 integrates functional features of both mammalian RIG‐I and MDA5, it can accommodate the two dsRNA ligands that they recognized. AlphaFold3 modeling yielded high‐confidence complexes of *Lc*MDA5 with the identical hairpin 5′ppp‐RNA duplex (ipTM: 0.83; pTM: 0.86) and the 15‐mer blunt‐ended dsRNA (ipTM: 0.84; pTM: 0.85) (Figure 4A). The overall architecture of the *Lc*MDA5‐5′ppp‐RNA duplex was similar to *Lc*MDA5‐blunt‐ended dsRNA complex. In both models, the two helicase domains (Hel1 and Hel2), the helicase insertion domain (Hel2i), the pincer domain, and the C‐terminal domain (CTD) form a ring that encircles the RNA stem (Figure 4A).

**FIGURE 4:**
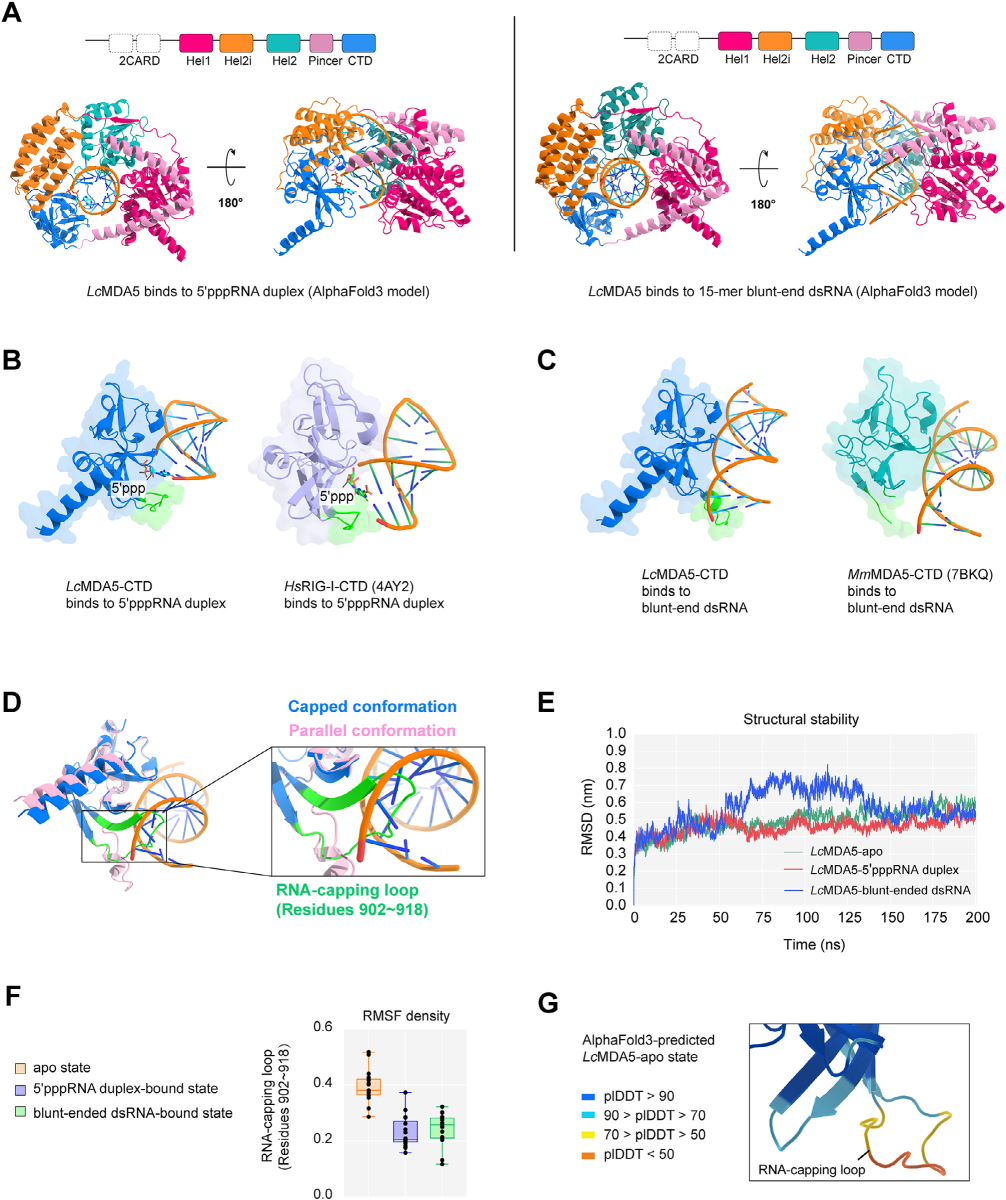
Structural insights into *Lc*MDA5 binding to 5′ppp‐RNA duplex or long dsRNA. (A) AlphaFold3‐predicted model of *Lc*MDA5 in complex with either a hairpin 5′ppp‐dsRNA or a 15‐mer blunt‐ended dsRNA. (B) Structural comparison of the CTDs of *Lc*MDA5 and human RIG‐I (*Hs*RIG‐I) bound to 5′ppp‐dsRNA. In both structures, the CTD loop rigidly caps the dsRNA terminus, enhancing stability. (C) Structural comparison of the CTDs of *Lc*MDA5 and murine MDA5 (*Mm*MDA5) bound to 15‐mer blunt‐ended dsRNA. The CTD loop in both proteins aligns parallel to the dsRNA axis. (D) The CTD conformation used for 5′ppp‐RNA duplex recognition is sterically incompatible with the (long) blunt‐ended dsRNA‐binding mode. (E) The RMSD of the protein and its dsRNA‐bound complexes was monitored over a 200 ns MD simulation to assess structural stability. (F) RMSF analysis of the RNA‐capping loop. MD simulations were performed for the apo, 5′ppp‐RNA duplex‐bound, and 15‐mer blunt‐ended dsRNA‐bound forms of *Lc*MDA5. RMSF values for the RNA‐capping loop were extracted and are presented as box plots. (G) Predicted pLDDT scores for the AlphaFold3‐predicted apo structure of *Lc*MDA5. The RNA‐capping loop exhibits pLDDT values below 50, suggesting intrinsic disorder in the unbound state.

The key structural difference was observed in the conformation of the CTD (Figure 4B, C). In the *Lc*MDA5‐5′ppp‐RNA duplex complex, the CTD capped the end of 5′ppp‐RNA duplex via the RNA‐capping loop (residues 902–918), forming a cavity that accommodates the 5′ppp moiety (Figure 4B). The predicted “capped” configuration resembles the dsRNA‐binding mode of human RIG‐I and is expected to prevent the 5′ppp‐RNA duplex from escaping the protein ring (Figure 4B). This finding is consistent with the previous biochemical evidence demonstrating that the CTD is essential for recognition of viral 5′ppp‐RNA by miiuy croaker MDA5 [18]. In predicted the *Lc*MDA5‐blunt‐ended dsRNA complex, the CTD is parallel to the dsRNA axis, adopting a conformation similar to that of mouse MDA5 (Figure 4C). This configuration permits long dsRNA to thread through the ring structure without steric hindrance [33]. The “capped” conformation observed in the 5′ppp‐RNA duplex‐bound structure is sterically incompatible with the passage of long dsRNA (Figure 4D).

To investigate the structural stability of *Lc*MDA5 in complex with either 5′ppp‐RNA duplex or blunt‐ended dsRNA, we performed molecular dynamics (MD) simulations of the AlphaFold3‐predicted apo form and two dsRNA‐bound forms of *Lc*MDA5. Structural stability was comparable for *Lc*MDA5 bound to 5′ppp‐RNA duplex or blunt‐ended dsRNA, as reflected by similar root mean square deviation (RMSD) values at the later stage of the simulations (Figure 4E). To evaluate local flexibility, we calculated root mean square fluctuation (RMSF) values from the MD trajectories. The RNA‐capping loop (residues 902–918) exhibited markedly reduced root mean square fluctuation (RMSF) values upon binding to either RNA ligand (Figure 4F, Figure S2A), indicating ligand‐induced structural stabilization of this region. Consistent with these findings, analysis of the AlphaFold3‐predicted apo structure of *Lc*MDA5 (pTM score: 0.92) revealed that the RNA‐capping loop has a notably low predicted local distance difference test (pLDDT) score (Figure 4G), suggesting that the region is flexible in the absence of dsRNA [34, 35]. To examine whether the 5′ppp‐RNA duplex stabilizes the “capping” conformation, we performed Normal Mode Analysis on the AlphaFold3‐predicted *Lc*MDA5‐5′ppp‐RNA duplex complex. When RNA was bound, the capping loop remained relatively rigid (Figure S2B). In contrast, removing the 5′ppp‐RNA duplex resulted in increased loop flexibility. These results suggest a ligand‐dependent transition from a flexible apo state to a stabilized conformation.

Together, these results indicate that flexibility of the RNA‐capping loop enables *Lc*MDA5 to bind either 5′ppp‐dsRNA or long dsRNA.

### 2.5 Characterization of Key *Lc*MDA5 CTD Residues Involved in 5′ppp‐dsRNA and Long dsRNA Binding

Our structural models revealed two distinct CTD interfaces for the respective ligands. In the 5′ppp‐RNA complex, residues Q907, Y918, C941, and K945 engage the 5′ppp group, while M885, H886, R887, H943, W962, and S963 stabilize the RNA backbone (Figure 5A). On the contrary, the *Lc*MDA5‐ blunt‐ended dsRNA complex involves both shared residues (M885, H886, H943, W962, S963) and additional contacts unique to this ligand (Y913, N916, R927, S930, K958) (Figure 5B). Based on dsRNA‐binding data from physiologically relevant LYCK cells (Figure 3A, B), we introduced mutations in the putative RNA‐binding sites of *Lc*MDA5 to investigate their effects on ligand binding and signaling. The impact of these mutations on dsRNA binding was analyzed in HEK293T cells lacking the endogenous RLRs [36, 37], to specifically evaluate the effects of individual mutations on *Lc*MDA5–RNA binding, while their effects on antiviral signaling were assessed in LYCK cells to preserve a physiologically relevant cellular context. Alanine substitution at residues H886, R887, Q907, Y918, H943, and K945 of *Lc*MDA5 significantly impaired its binding to 5′ppp‐dsRNA and attenuated the associated downstream signaling response (Figure 5C, D). In contrast, substitutions at residues H886, H943, and K958 impaired poly(I:C)‐HMW binding and reduced downstream signaling activity (Figure 5E, F). Critically, mutation at three residues (Q907, Y918, and K945) involved in *Lc*MDA5 binding to the 5′ppp moiety did not affect its recognition of poly(I:C)‐HMW (Figure 5F), indicating that these residues specifically engage the 5′ppp moiety rather than the dsRNA stem. To assess the physiological relevance of this recognition mode, we examined the role of *Lc*MDA5 during infection with vesicular stomatitis virus (VSV), a well‐established RIG‐I‐recognized model virus [38]. VSV possesses a broad host range and is also capable of infecting LYCK cells (Figure S3A) [39]. Consistent with the ability of *Lc*MDA5 to recognize 5′ppp‐containing RNA, VSV‐derived RNA induced *Lc*MDA5 oligomerization, although less efficiently than poly(I:C)‐HMW (Figure S3B). Treatment of VSV RNA with calf intestinal alkaline phosphatase (CIAP) abolished its ability to induce *Lc*MDA5 oligomerization (Figure S3B), indicating that terminal phosphate groups are required for viral RNA‐induced *Lc*MDA5 oligomerization. Furthermore, rescue experiments in *Lc*MDA5‐knockdown cells demonstrated that mutants defective in 5′ppp‐dsRNA recognition exhibited impaired antiviral activity compared with wild‐type *Lc*MDA5, as evidenced by increased viral titers and reduced induction of type I IFNs (Figure S3C, D). These results indicate that 5′ppp‐dsRNA recognition by *Lc*MDA5 contributes to its antiviral defense against VSV infection.

**FIGURE 5:**
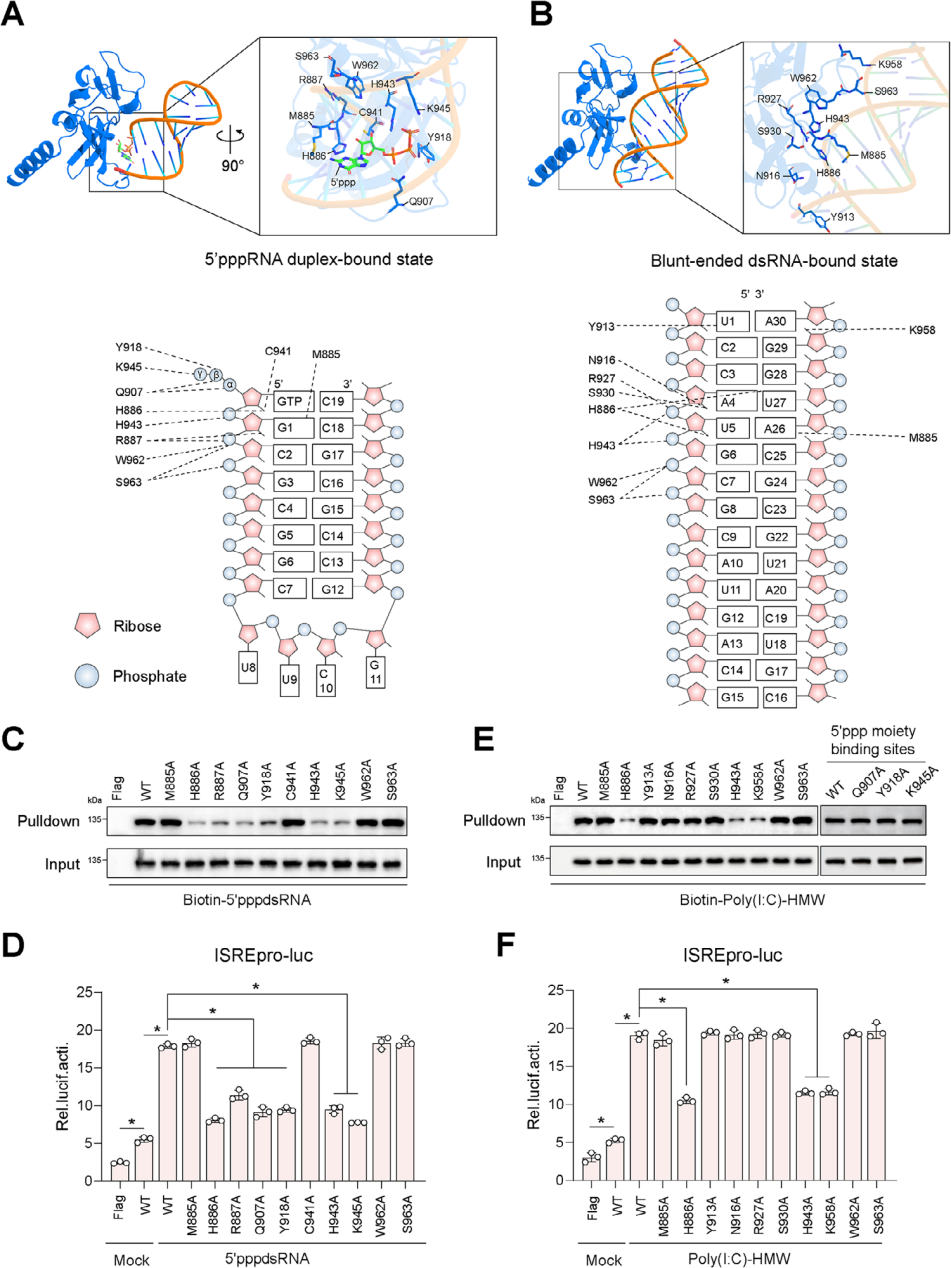
Characterization of key residues within the *Lc*MDA5 CTD crucial for 5′ppp‐dsRNA and long dsRNA binding. (A) Structural representation of the *Lc*MDA5 CTD showing key amino acid residues involved in 5′ppp‐RNA duplex recognition (top), with a schematic depiction of protein‐RNA interactions (bottom). (B) Structural representation of *Lc*MDA5 CTD residues mediating blunt‐ended 15‐mer dsRNA binding (top), with a schematic diagram of the protein‐RNA interface (bottom). (C) RNA‐binding capacity of wild‐type (WT) and mutant *Lc*MDA5 proteins for 5′ppp‐dsRNA. Biotinylated 5′ppp‐dsRNA was incubated with HEK293T cell lysates expressing the indicated constructs. RNA–protein complexes were pulled down using streptavidin beads and subjected to Western blot analysis. (D) Functional analysis of *Lc*MDA5‐WT and point mutants following 5′ppp‐dsRNA transfection. LYCK cells were co‐transfected with plasmids expressing either WT or mutant *Lc*MDA5, an ISRE‐driven firefly luciferase reporter, and pRL‐TK Renilla luciferase plasmid. After 24 h, cells were transfected with 5′ppp‐dsRNA for an additional 24 h, after which luciferase activity was measured to assess ISRE‐driven promoter activation. Data represent means ± SD from three independent experiments (*p < 0.05, one‐way ANOVA followed by Bonferroni post hoc tests). (E) Binding of WT and mutant *Lc*MDA5 proteins to poly(I:C)‐HMW. Biotinylated poly(I:C)‐HMW was incubated with HEK293T cell lysates expressing the indicated constructs, and RNA‐protein complexes were analyzed as described in (C). (F) Functional analysis of WT and mutant *Lc*MDA5 in response to poly(I:C)‐HMW transfection. LYCK cells were co‐transfected with plasmids expressing either WT or mutant *Lc*MDA5, an ISRE‐driven firefly luciferase reporter, and the pRL‐TK Renilla luciferase plasmid. After 24 h, poly(I:C)‐HMW was transfected for an additional 24 h. Luciferase activity was then measured to assess ISRE‐driven promoter activation. Data represent means ± SD from three independent experiments (*p < 0.05, one‐way ANOVA followed by Bonferroni post hoc tests).

Collectively, these findings demonstrate that the CTD of *Lc*MDA5 is essential for the recognition of both canonical RIG‐I and MDA5 ligands through distinct binding configurations.

### 2.6 Both *Lc*MAVS and *Lc*STING Are Required for *Lc*MDA5‐Mediated Signal Transduction

In mammals, RIG‐I engages both STING and MAVS for signal transduction, whereas MDA5 signals exclusively via MAVS [6]. To identify the adaptors through which *Lc*MDA5 transmits signaling, we conducted co‐immunoprecipitation (co‐IP) assays in LYCK cells transfected with plasmids expressing epitope‐tagged proteins. Both *Lc*STING and *Lc*MAVS co‐precipitated with *Lc*MDA5, indicating that *Lc*MDA5 associates with both adaptors (Figure 6A). Confocal microscopy further confirmed that *Lc*MDA5 co‐localized with both *Lc*STING and *Lc*MAVS in HEK293T and LYCK cells (Figure 6B). Additionally, either *Lc*STING or *Lc*MAVS enhanced *Lc*MDA5‐induced promoter activity of two type I IFNs (*Lc*IFNi and *Lc*IFNh) (Figure 6C), supporting their roles as adaptor proteins for *Lc*MDA5. To validate the involvement of *Lc*STING and *Lc*MAVS in type I IFN induction triggered by 5′ppp‐dsRNA or poly(I:C)‐HMW, we individually knocked down *Lc*STING or *Lc*MAVS in LYCK cells. Knockdown analyses confirmed that depletion of *Lc*STING did not affect *Lc*MAVS expression, and vice versa, ensuring that the independent contribution of each adaptor in the LYCK cell model could be evaluated (Figure 6D). Protein‐level knockdown of *Lc*STING and *Lc*MAVS was further validated by Western blot (Figure S4). Subsequent experiments indicated that silencing either adaptor inhibited endogenous *Lc*TBK1 and *Lc*IRF3 phosphorylation (Figure 6E, F), consequently reducing the transcriptional induction of type I IFNs in response to both dsRNA ligands (Figure 6G–J).

**FIGURE 6:**
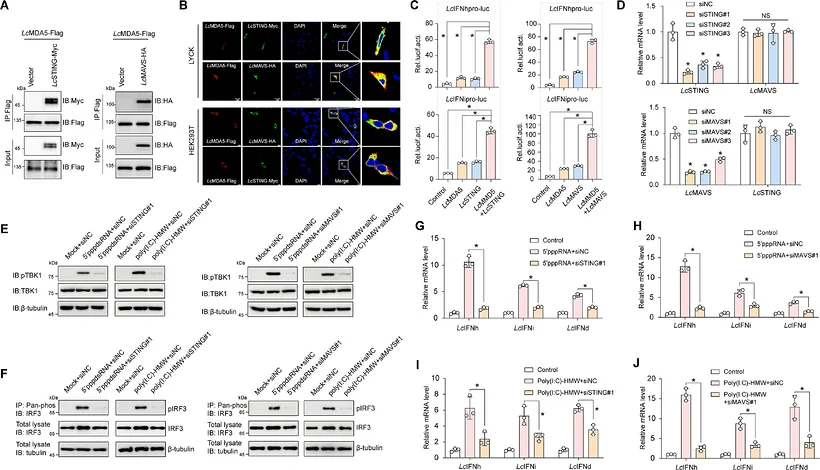
*Lc*STING and *Lc*MAVS contribute to *Lc*MDA5‐mediated signal transduction. (A) *Lc*MDA5 associates with both *Lc*MAVS and *Lc*STING. LYCK cells were transfected with 5 µg of the indicated epitope‐tagged plasmids for 24 h. Cell lysates were subjected to immunoprecipitation using antibody‐conjugated magnetic beads, and protein interactions were detected by Western blot with tag‐specific antibodies. (B) *Lc*MAVS and *Lc*STING co‐localize with *Lc*MDA5. HEK293T or LYCK cells were co‐transfected with Flag‐tagged *Lc*MDA5 and either Myc‐tagged *Lc*STING or HA‐tagged *Lc*MAVS. Cells were fixed, immunostained using the appropriate antibodies, and visualized via confocal immunofluorescence microscopy. Nuclei were stained with DAPI (blue). Yellow signals in the merged images indicate co‐localization. Scale bar, 10 µm. (C) Both *Lc*STING and *Lc*MAVS enhance *Lc*MDA5‐mediated activation of type I IFN promoters. EPC cells were co‐transfected with plasmids expressing *Lc*MDA5 and either *Lc*STING or *Lc*MAVS, along with luciferase reporter constructs driven by the *Lc*IFNh or *Lc*IFNi promoters. Luciferase activity was measured 24 h post‐transfection to evaluate promoter activation. (D) Efficient knockdown of *Lc*STING or *Lc*MAVS was confirmed by RT‐qPCR. LYCK cells were transfected with 100 nM siRNA targeting *Lc*STING or *Lc*MAVS, or a non‐targeting control siRNA (siNC). Total RNA was extracted 24 h post‐transfection to assess knockdown efficiency. (E, F) Knockdown of *Lc*STING or *Lc*MAVS impairs phosphorylation of *Lc*TBK1 (E) and *Lc*IRF3 (F) in response to 5′ppp‐dsRNA or poly(I:C)‐HMW stimulation (by transfection). LYCK cells were transfected with siRNAs targeting *Lc*STING (100 nM) or *Lc*MAVS (100 nM) for 24 h, followed by 24 h stimulation with either 5′ppp‐dsRNA (2.5 µg/mL) or poly(I:C)‐HMW (2.5 µg/mL). Phosphorylation of *Lc*IRF3 and *Lc*TBK1 was then assessed using a pan‐phosphoserine/threonine antibody and a phospho‐specific TBK1 antibody, respectively. (G–J) Knockdown of either *Lc*STING or *Lc*MAVS reduced the transcription of type I IFNs induced by 5′ppp‐dsRNA (G and H) or poly(I:C)‐HMW (I and J). Data represent the mean ± SD from three independent experiments. Asterisks indicate statistical significance (*p* < 0.05, two‐tailed unpaired Student's *t*‐test); NS, not significant.

### 2.7 *Lc*STING Operates Downstream of *Lc*MAVS in the *Lc*MDA5‐Mediated Signaling Pathway

The findings above demonstrate that both *Lc*STING and *Lc*MAVS are required for *Lc*MDA5‐dependent type I IFN signaling, which prompted us to investigate the interaction between these two adaptors. Immunofluorescence and co‐IP assays revealed that *Lc*STING co‐localized and interacted with *Lc*MAVS (Figure 7A, B). Through both ectopic expression in HEK293T cells and endogenous co‐IP in LYCK cells, we confirmed that *Lc*MDA5, *Lc*MAVS, and *Lc*STING co‐existed within the same signaling complex (Figure 7C, D). Within this complex, *Lc*MAVS appeared to bridge the signal, as its overexpression enhanced the *Lc*MDA5–*Lc*STING interaction (Figure S5A), consistent with findings in RIG‐I‐deficient avian systems where MAVS similarly facilitates the interaction between MDA5 and STING [40]. This assembly is strictly dependent on the N‐terminal caspase activation and recruitment domain CARDs of *Lc*MDA5. The binding of MDA5 with MAVS is known to be mediated by CARD–CARD interactions [41, 42]. Deletion of the two CARDs of *Lc*MDA5 abolished its interaction with *Lc*MAVS and simultaneously eliminated its association with *Lc*STING in LYCK cells (Figure S5B).

**FIGURE 7:**
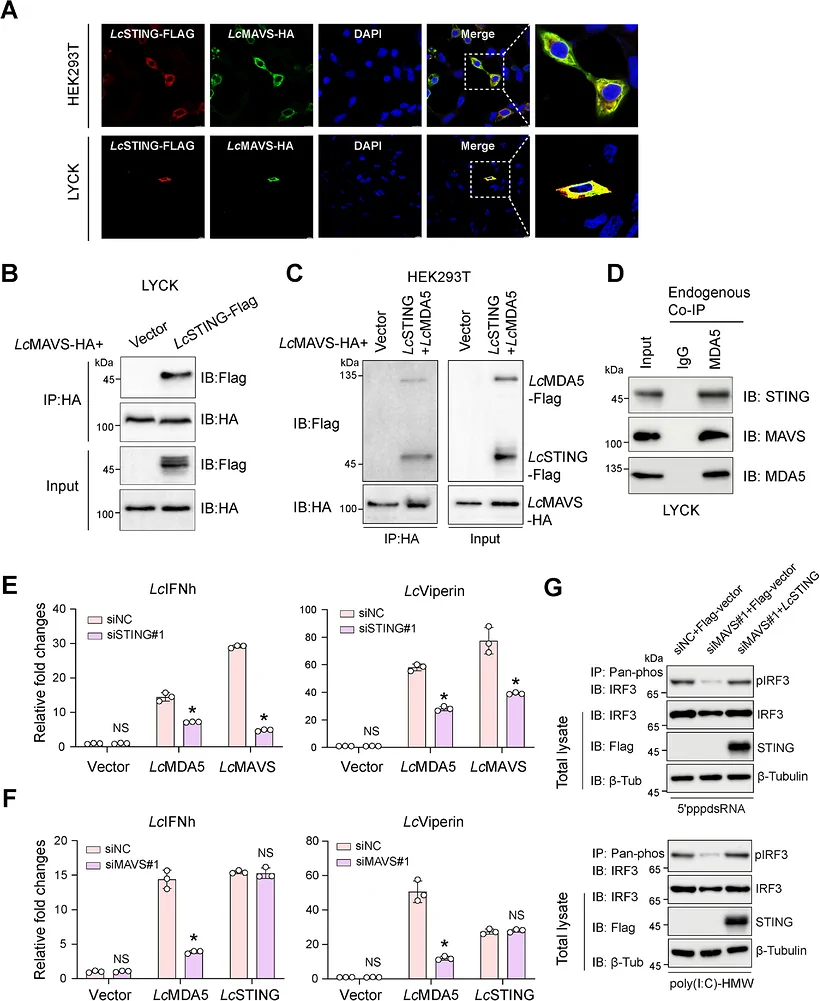
*Lc*STING operates downstream of *Lc*MAVS in the *Lc*MDA5‐mediated signaling pathway. (A) *Lc*STING co‐localizes with *Lc*MAVS in HEK293T and LYCK cells. Cells were co‐transfected with FLAG‐tagged *Lc*STING and HA‐tagged *Lc*MAVS, and imaged using confocal microscopy. Scale bar: 10 µm. (B) *Lc*STING associates with *Lc*MAVS. LYCK cells were transfected with 5 µg of the indicated plasmids for 24 h, followed by co‐IP and Western blot analysis. (C) *Lc*MDA5, *Lc*STING, and *Lc*MAVS are present within the same complex. HEK293T cells were co‐transfected with Flag‐tagged *Lc*MDA5, Flag‐tagged *Lc*STING, and HA‐tagged *Lc*MAVS expression plasmids. After 24 h, lysates were subjected to immunoprecipitation and analyzed by Western blot. (D) Endogenous association among *Lc*MDA5, *Lc*STING, and *Lc*MAVS in LYCK cells. Cell lysates were immunoprecipitated with an anti‐MDA5 antibody and subsequently analyzed by Western blot with anti‐STING and anti‐MAVS antibodies to detect protein interaction. (E) *Lc*STING knockdown reduces *Lc*MDA5‐ or *Lc*MAVS‐induced type I IFN transcriptional responses. LYCK cells were transfected with siSTING or control siRNA (siNC). After 24 h, cells were stimulated by the overexpression of either *Lc*MDA5 or *Lc*MAVS. Target gene expression was quantified by RT‐qPCR. (F) *Lc*MAVS knockdown reduces *Lc*MDA5‐ but not *Lc*STING‐induced transcriptional activation. LYCK cells were transfected with siRNA targeting *Lc*MAVS (siMAVS) or control siRNA (siNC). After 24 h, cells were stimulated by overexpression of either *Lc*MDA5 or *Lc*STING, and expression of downstream genes was measured by RT‐qPCR. (G) Overexpression of *Lc*STING rescues impaired *Lc*IRF3 phosphorylation caused by *Lc*MAVS knockdown. LYCK cells were co‐transfected with *Lc*MAVS‐targeting siRNA (100 nM) and Flag‐tagged *Lc*STING plasmid for 24 h, followed by stimulation with 5′ppp‐dsRNA or poly(I:C)‐HMW (both at 2.5 µg/mL) for another 24 h. Phosphorylation of *Lc*IRF3 was then assessed using a pan‐ phosphoserine/threonine antibody. Data represent the mean ± SD from three independent experiments. Asterisks indicate statistical significance (*p < 0.05, two‐tailed unpaired Student's *t*‐test).

Subsequent knockdown experiments showed that knockdown of *Lc*STING suppressed both *Lc*MDA5‐ and *Lc*MAVS‐overexpression‐induced expression of *Lc*IFNh and *Lc*Viperin (Figure 7E), indicating that *Lc*STING is required for signaling triggered by the upstream sensor *Lc*MDA5 or the adaptor *Lc*MAVS. Conversely, knockdown of *Lc*MAVS impaired *Lc*MDA5‐overexpression‐induced expression of *Lc*IFNh and *Lc*Viperin but did not affect their expression induced by *Lc*STING overexpression (Figure 7F). All these results suggest that *Lc*STING acts downstream of *Lc*MAVS within the *Lc*MDA5‐initiated signaling pathway. To validate this hierarchical relationship, we performed rescue experiments based on *Lc*IRF3 phosphorylation status. Having established that *Lc*MAVS knockdown impairs ligand‐induced *Lc*IRF3 phosphorylation (Figure 6F), we reasoned that if *Lc*STING indeed functions downstream of *Lc*MAVS, its ectopic expression should rescue this signaling defect. Supporting this hypothesis, while *Lc*MAVS silencing expectedly abolished *Lc*IRF3 phosphorylation in response to 5′ppp‐dsRNA or poly(I:C)‐HMW, overexpression of *Lc*STING successfully restored *Lc*IRF3 phosphorylation (Figure 7G). These rescue results strongly indicate that *Lc*STING operates downstream of *Lc*MAVS in the *Lc*MDA5‐mediated signaling pathway.

## 3 Discussion

In the present study, we demonstrate that MDA5 in a RIG‐I‐deficient teleost functions as a dual‐ligand RNA sensor capable of recognizing both 5′ppp‐dsRNA and long dsRNA, which is typically attributed to the independent recognition of RIG‐I and MDA5. Furthermore, the binding of MDA5 to 5′ppp‐dsRNA or long dsRNA activated the antiviral signals through a unified MAVS–STING–TBK1/IRF3 signaling axis. These findings suggest a flexible architecture of vertebrate cytosolic RNA sensing in which a single RLR integrates ligand‐recognition and signaling properties classically partitioned between RIG‐I and MDA5.

Teleost RLR signaling appears to exhibit a greater degree of functional overlap between RIG‐I and MDA5 than that reported in mammals. For example, zebrafish RIG‐I and MDA5 both interact with MAVS and exhibit antiviral activity against spring viremia of carp virus [43]. In grass carp (*Ctenopharyngodon idella*), RIG‐I and MDA5 inhibit grass carp reovirus infection, although MDA5 induces a stronger type I IFN response than RIG‐I during infection [17]. Unlike mammalian STING, which transmits RIG‐I‐activated signaling but not MDA5, STING in crucian carp (*Carassius auratus L*.) mediates type I IFN production elicited by both RIG‐I and MDA5 [13]. These observations suggest that functional boundary between RIG‐I and MDA5 may be less distinct in teleosts than in mammals. Consistently, zebrafish MDA5 associated with both 5′ppp‐dsRNA and long dsRNA in an in vitro RNA pull‐down assay (Figure S6), suggesting that functional overlap between RIG‐I and MDA5 may exist in some teleost lineages regardless of RIG‐I status. Geng et al. proposed a model in which functional overlap between MDA5 and RIG‐I may have contributed to the loss of RIG‐I during vertebrate evolution, as teleost MDA5 can recognize ligands associated with RIG‐I [18]. This hypothesis is consistent with the evolutionary principle that the presence of overlapping functions or alternative pathways can reduce the fitness consequences of gene loss [19]. Given that RIG‐I loss has occurred independently at least three times during teleost diversification, the evolutionary histories of teleost RLR systems are likely to be complex, and establishing a unified evolutionary model remains challenging [12]. Nevertheless, our findings in *L. crocea* provide evidence of functional plasticity within the teleost RLR family.

Mammalian RIG‐I CTD can cap the end of dsRNA via a loop, which is critical for recognizing the 5′ppp moiety [3]. The corresponding loop in mammalian MDA5 does not appear to contribute substantially to dsRNA binding, as truncation of this region does not affect MDA5 binding to dsRNA [44, 45]. The conformation of the RNA‐binding loop determines the sensitivity of RLRs to dsRNA [45]. Our structural model predicts that the *Lc*MDA5 CTD contains a loop configuration that may facilitate accommodation of the 5′ppp‐RNA terminus. In contrast, modeling of mouse MDA5 with 5′ppp‐dsRNA did not predict a comparable CTD configuration (Figure S7). When *Lc*MDA5 binds to blunt‐ended 15‐mer dsRNA, the CTD interacts with the dsRNA stem, with the loop parallel to the dsRNA ligand. Mutation of three residues (Q907, Y918, and K945) involved in 5′ppp binding, did not affect *Lc*MDA5 recognition of poly(I:C)‐HMW. The RNA‐capping loop exhibited substantially lower pLDDT values than the surrounding RNA‐binding interface. According to current interpretations of AlphaFold confidence metrics, low pLDDT values often indicate conformational flexibility or intrinsic disorder [34, 35]. Accordingly, the RNA‐capping loop is likely to be conformationally flexible. MD simulations and normal mode analyses further suggested greater loop mobility in the absence of RNA and increased stabilization in the predicted ligand‐bound state. Such flexible loop regions are often poorly resolved in experimentally determined structures due to conformational heterogeneity. The AlphaFold3 models, MD simulations, and mutational analyses support a model in which RNA binding stabilizes the RNA‐capping loop and facilitates recognition of 5′ppp‐dsRNA. Engineered dsRNA molecules optimized for MDA5 activation could function as immunological adjuvants and may have potential applications in vaccine development for aquaculture. However, whether other domains also contribute to ligand selectivity remains to be investigated.

In miiuy croaker, MDA5 senses both long dsRNA and 5′pppRNA from *Siniperca chuatsi* rhabdovirus (SCRV), and SCRV‐induced IFN‐1 production is impaired upon STING silencing [18]. However, how MDA5 mediates signaling pathways in response to different dsRNA ligands remains unclear (5′ppp‐dsRNA vs. long dsRNA). We show that both *Lc*STING and *Lc*MAVS are required for *Lc*MDA5‐dependent type I IFN production in response to cytosolic 5′ppp‐dsRNA or poly(I:C)‐HMW. Knockdown of *Lc*STING suppressed type I IFN and ISG induction by both *Lc*MDA5 and *Lc*MAVS overexpression, whereas knockdown of *Lc*MAVS impaired induction by *Lc*MDA5 but not by *Lc*STING overexpression, indicating that *Lc*STING functions downstream of *Lc*MAVS within the *Lc*MDA5‐initiated signaling pathway. *Lc*MAVS knockdown impaired IRF3 phosphorylation induced by either 5′ppp‐dsRNA or poly(I:C)‐HMW, yet overexpression of *Lc*STING in *Lc*MAVS‐silenced cells restored IRF3 phosphorylation (Figure 7G), further supporting a model in which *Lc*MDA5 recognizes both 5′ppp‐dsRNA and long dsRNA and signals through an integrated MAVS‐STING‐TBK1/IRF3 axis. Interestingly, MDA5‐STING coupling has also been reported in other RIG‐I‐deficient vertebrates, including chickens and tree shrews [40, 46]. Considering that RLR family members are thought to have diverged prior to the emergence of cnidarians [47], the independent emergence of MDA5‐triggered STING‐dependent signaling in distinct lineages may represent a convergent evolutionary example.

Compared with mammalian STING, teleost STING possesses distinct structural and signaling features that may underlie their divergent functions in antiviral immunity. In particular, the C‐terminal tail of teleost STING contains TRAF‐binding motifs that mediate its association with TNF receptor‐associated factor 6 (TRAF6) [48], a key signaling adaptor involved in MAVS‐mediated antiviral responses [49]. This feature may allow teleost STING to contribute directly to RLR signaling. For example, Grass Carp Reovirus VP41 targets fish STING to inhibit RLR‐mediated IFN induction [50]. Furthermore, a dominant‐negative STING mutant in crucian carp abolishes type I IFN activation mediated by both RIG‐I and MDA5 [13]. Our findings further demonstrated that *Lc*STING is essential for the *Lc*MDA5‐mediated signaling pathway. Thus, STING appears to play a more prominent role in antiviral responses to RNA viruses in teleosts than in mammals.

In conclusion, we identify *Lc*MDA5 as a dual‐specificity cytosolic RNA sensor capable of recognizing both 5′ppp‐dsRNA and long dsRNA. Biochemical, mutational, and structural analyses support our proposed model, in which conformational flexibility within the CTD enables *Lc*MDA5 to adopt alternative ligand‐recognition modes, allowing the CTD to cap the 5′ppp‐dsRNA terminus for RIG‐I‐like recognition or orienting the CTD parallel to long‐dsRNA axis for canonical MDA5 recognition. Downstream, *L*cMDA5 activates an integrated MAVS–STING–TBK1/IRF3 signaling cascade, with STING functioning downstream of MAVS to promote type I IFN responses (Figure 8). Together, these findings reveal that the functional specialization of vertebrate RLRs is less rigid than inferred from mammalian systems and show how a single RNA sensor can integrate recognition and signaling properties classically distributed between RIG‐I and MDA5. Such receptor plasticity provides a mechanistic framework for understanding how robust antiviral sensing can be maintained across vertebrate lineages despite evolutionary loss of individual innate immune receptors.

**FIGURE 8:**
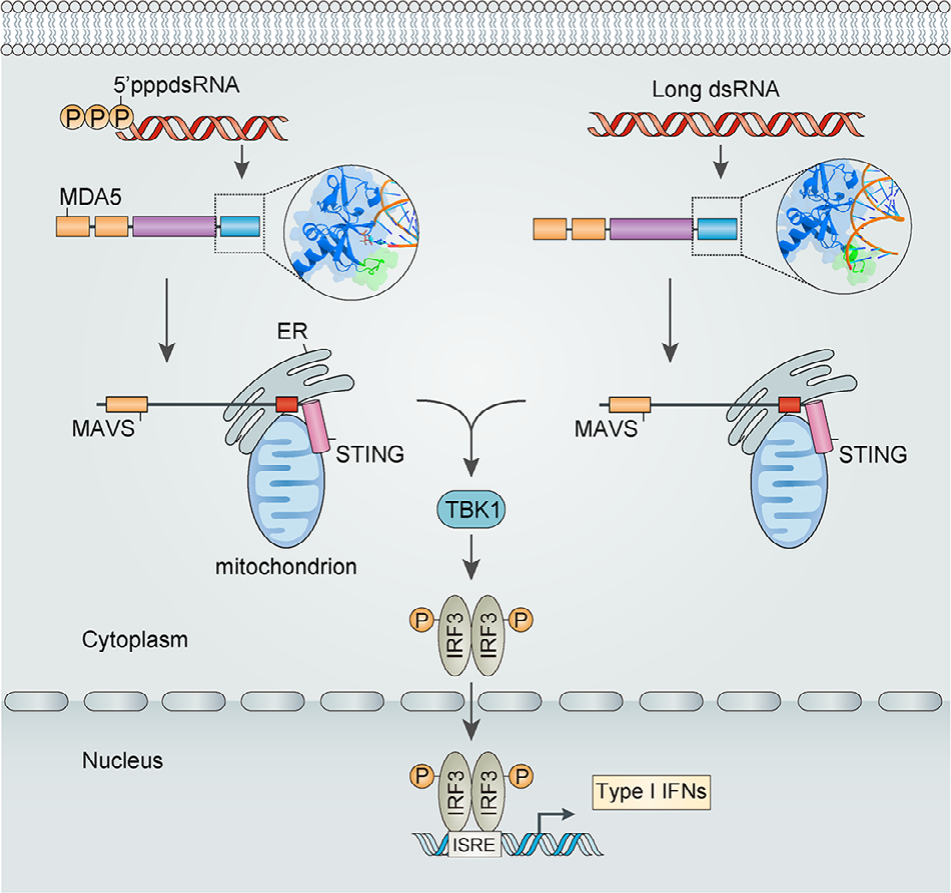
MDA5 activates an integrated MAVS–STING–TBK1/IRF3 signaling cascade in the large yellow croaker (*Larimichthys crocea*). Intracellular 5′ppp‐dsRNA or long dsRNA are recognized by MDA5. Upon recognition, MDA5 activates downstream signaling via the MAVS–STING pathway, leading to the phosphorylation of IRF3. Phosphorylated IRF3 then translocates to the nucleus, where it binds to IFN‐stimulated response elements (ISREs) in type I IFN gene promoters, which promotes the production of type I IFNs.

## 4 Materials and Methods

### 4.1 Cells, Antibodies, and Reagents

Human embryonic kidney 293T cells (HEK293T, ATCC CRL‐3216) were cultured in Dulbecco's Modified Eagle Medium (DMEM; Gibco, Cat#11965092) supplemented with 10% fetal bovine serum (FBS) and 1× penicillin–streptomycin (Gibco, Cat#10378016), and maintained in a humidified incubator at 37°C with 5% CO_2_. EPC cells (ATCC CRL‐2872) were cultured in Medium 199 (M199; Gibco, Cat#11150067) supplemented with 10% FBS and 1× penicillin–streptomycin, and maintained in a humidified incubator at 27°C with 5% CO_2_. LYCK cells (RRID: CVCL_HP90), previously established in our laboratory [51], were cultured in Leibovitz's L‐15 medium (Gibco, Cat#11415064) supplemented with 10% FBS and 1× penicillin–streptomycin, and maintained at 27°C under atmospheric conditions without CO_2_ supplementation. The LYCK cells were authenticated by chromosomal analysis, indicating a modal diploid chromosome number of 48, matching that of the *L. crocea* kidney [51]. Zebrafish embryonic fibroblast cells (ZF4, ATCC CRL‐2050) were cultured in DMEM/F‐12 medium (Gibco, Cat#11330032) supplemented with 10% FBS and 1× penicillin‐streptomycin, and maintained in a humidified incubator at 27°C with 5% CO_2_. All cell lines were regularly tested to be free of mycoplasma (Mycoplasma PCR Detection Kit, Beyotime, cat#C0301S).

Antibodies used for co‐IP and immunoblotting (Western blot) were as follows: Flag rabbit monoclonal antibody (Cell Signaling, Cat#14793, 1:1,000); Myc rabbit monoclonal antibody (Abclonal, Cat#AE070, 1:1,000); HA rabbit monoclonal antibody (Cell Signaling, Cat#3724, 1:1,000); β‐tubulin mouse monoclonal antibody (Abmart Cat# M20005, 1:1000); horseradish peroxidase (HRP)‐conjugated goat anti‐mouse/rabbit IgG secondary antibodies (Proteintech, Cat#SA00001‐1 and Cat#SA00001‐2, 1:10,000); VSV‐G rabbit polyclonal antibody (Thermo Scientific, Cat#PA1‐30138); pan‐phospho‐serine/threonine mouse antibody (ABclonal, Cat# AP1067, 5 µg per 30 µL beads); TBK1 rabbit polyclonal antibody (Boster, Cat# BM4038, 1:1,000); Phospho‐TBK1 rabbit monoclonal antibody (Cell Signaling Technology, Cat#5483, 1:1000) and STING rabbit polyclonal antibody (Abcam, Cat#ab288157). Antibodies used for immunofluorescence were as follows: Flag mouse monoclonal antibody (Proteintech, Cat#66008‐4‐Ig, 1:200); HA rabbit polyclonal antibody (Proteintech, Cat#51064‐2‐AP, 1:200); Myc rabbit polyclonal antibody (Proteintech, Cat#16286‐1‐AP, 1:200); CoraLite594‐conjugated secondary antibody (Proteintech, Cat#SA00013‐3, 1:200); and CoraLite488‐conjugated secondary antibody (Proteintech, Cat#SA00013‐2, 1:200). Polyclonal antibodies against *Lc*MDA5 were prepared as described in a previous study [52], and *Lc*MAVS was prepared by AtaGenix Laboratories Co, Ltd. Polyclonal antibody against *Lc*IRF3 was prepared as described in a previous study [53].

Poly(I:C)‐HMW (1.5‐8 kb, InvivoGen, Cat#tlrl‐pic), Poly(I:C)‐HMW‐Biotin (InvivoGen, Cat#tlrl‐picb), 5′ppp‐dsRNA (InvivoGen, Cat#tlrl‐3prna), 5′ppp‐dsRNA Control (InvivoGen, Cat#tlrl‐3prnac; same sequence as 5′ppp‐dsRNA but lacking the 5′ppp moiety) and R848 (InvivoGen, Cat#tlrl‐r848‐1) were used as ligands in this study. EZ‐Link Psoralen‐PEG3‐Biotin was purchased from Thermo Scientific (Cat#29986). For plasmid transfection, Hieff Trans Liposomal Transfection Reagent (Yeasen, Cat# 40802ES03) was used for LYCK and ZF4 cells, while Lipofectamine 3000 (Thermo Scientific) was used for HEK293T and EPC cells. Hieff Trans Liposomal Transfection Reagent (Yeasen, Cat# 40802ES03) was utilized to perform both single siRNA transfections and co‐transfections with plasmid DNA. For cytoplasmic delivery of RNA ligands, transfections were performed using LyoVec (InvivoGen, Cat#lyec‐1). Viral RNA transfection was performed using Lipofectamine 3000 (Thermo Scientific).

### 4.2 Plasmid Construction

The expression plasmid encoding *Lc*MDA5 (GenBank: PX573165) was constructed by cloning its open reading frame (ORF) into the p3xFLAG‐CMV‐14 (Sigma‐Aldrich) vector. An N‐terminal CARD‐deletion mutant of *Lc*MDA5 (*Lc*MDA5‐ΔCARD; residues 1–294 deleted) was generated by cloning the truncated ORF into the same vector. The *Lc*MAVS (XM_010756308.3) expression plasmid was generated by inserting its ORF into the pCMV‐HA‐C (Clontech) vector. Plasmids for Flag‐ and Myc‐tagged *Lc*STING (XM_010732873.3) expression were constructed by cloning the ORF into the p3xFLAG‐CMV‐14 and pcDNA3.1/myc‐His(+) A (Sigma‐Aldrich) vectors, respectively. The ORF of *Dr*MDA5 (NM_001308563.1) was cloned into p3xFLAG‐CMV‐14. The *Lc*IFNhpro‐luc reporter plasmid was described previously [53]. The *Lc*IFNipro‐luc construct was generated by inserting the promoter region (‐567 – +171; PX687857) of the *Lc*IFNi gene into the pGL3‐basic vector. The IFN‐stimulated response element (ISRE)‐driven firefly luciferase reporter plasmid was purchased from Addgene. Site‐directed mutant plasmids of *Lc*MDA5 were produced by Tsing Ke Biotechnology Co., Ltd. (Beijing, China) using the wild‐type *Lc*MDA5 plasmid as a template.

### 4.3 RNA Interference and Real‐Time qPCR (RT‐qPCR)

Synthetic siRNAs targeting *Lc*MDA5 (NCBI ID: 104920059), *Lc*TLR3 (NCBI ID: 104917775), *Lc*TLR7 (NCBI ID: 104928705), *Lc*MAVS (NCBI ID: 104939758), and *Lc*STING (NCBI ID: 104920568) were designed and synthesized by Tsingke Biotechnology Co., Ltd. (Beijing, China). The sequences of the siRNAs were provided in Table S1. Total RNA was extracted using TRIzol Reagent (Invitrogen) and reverse transcribed into first‐strand cDNA using the HiScript III first Strand cDNA Synthesis Kit with gDNA wiper (Vazyme Biotech). RT‐qPCR was performed using a QuantStudio 5 Real‐Time PCR System (Applied Biosystems). Gene expression levels were calculated using the 2^−ΔΔCT^ method, with β‐actin serving as the reference gene. The specificity of primers for the target genes was validated in previous studies [53, 54, 55, 56, 57].

### 4.4 Luciferase Activity Assay

The luciferase reporter assay was conducted as previously described, with slight modifications [52]. To assess the signaling activity of *Lc*MDA5, LYCK cells seeded in 24‐well plates (2.5 × 10^5^ cells/well) were co‐transfected with 25 ng of pRL‐TK, 250 ng of an ISRE‐driven firefly luciferase reporter plasmid, and 250 ng of either wild‐type or mutant *Lc*MDA5 expression plasmids or the empty vector. After 24 h, cells were transfected with either 5′ppp‐dsRNA (2.5 µg/mL) or poly(I:C)‐HMW (2.5 µg/mL), with mock‐transfected cells serving as controls. Following an additional 24 h incubation, cells were washed with ice‐cold PBS and lysed in Passive Lysis Buffer (Promega). Relative luciferase activity was quantified as the ratio of firefly to Renilla luciferase luminescence. To evaluate the potential synergistic effects of *Lc*MDA5 with either *Lc*STING or *Lc*MAVS, EPC cells (2.5 × 10^5^ cells/well) were co‐transfected with 25 ng of pRL‐TK, 250 ng of an IFN‐responsive firefly luciferase reporter plasmid, and 250 ng of the respective expression plasmids. An empty vector was used to equalize the total amount of transfected DNA. After 24 h of transfection, relative luciferase activity was determined as the ratio of firefly to Renilla luciferase signals.

### 4.5 Viral Infection

VSV was gifted by Professor Mingxian Chang at the Institute of Hydrobiology, Chinese Academy of Sciences. Virus stocks were propagated and titrated using a TCID50 assay in HEK293T cells. LYCK cells were infected with VSV at a multiplicity of infection (MOI) of 0.01. After a 60 min adsorption period, infected cells were incubated and harvested at 12 h post‐infection. Viral RNA was prepared as previously described, with minor modifications [58]. Briefly, HEK293T cells were infected with VSV at an MOI of 0.01 for 12 h, and total RNA was extracted using TRIzol Reagent according to the manufacturer's instructions. RNA dephosphorylation was performed by incubating 10 µg of RNA with 10 U of calf intestinal alkaline phosphatase (CIAP, Thermo Fisher, Cat#18009019) in a 50 µL reaction mixture at 37°C for 1 h. The reaction was terminated by heat inactivation of CIAP at 65°C for 15 min. The dephosphorylated RNA was purified by phenol‐chloroform extraction and ethanol precipitation prior to transfection.

### 4.6 Semi‐denaturating Detergent Agarose Gel Electrophoresis

LYCK cells were lysed in NP40 lysis buffer at 4°C for 30 min. Cell lysates were clarified by centrifugation at 16,000 × g for 10 min at 4°C. The clarified lysates were then mixed with 4× SDD‐AGE loading buffer (2× TAE buffer, 20% glycerol, 8% SDS) and incubated at room temperature for 15 min. Samples were separated on a 1.7% agarose gel containing 0.1% SDS and electrophoresed in TAE running buffer supplemented with 0.1% SDS at 25 V for 16 h at 4°C. Proteins were transferred onto a PVDF membrane and analysed byimmunoblotting (Western blot) with the indicated antibodies.

### 4.7 RNA Pull‐Down

5′ppp‐dsRNA or its negative control dsRNA was biotin‐labeled using EZ‐Link Psoralen‐PEG3‐Biotin (Thermo Scientific, Cat#29986) under 365 nm UV light. Biotinylated poly(I:C)‐HMW was purchased from InvivoGen (Cat#tlrl‐picb). LYCK or HEK293T cells were seeded in 10‐cm dishes (5 × 10^6^ cells/dish). For analyses involving exogenously expressed proteins, LYCK or HEK293T cells were transfected with 10 µg of the indicated plasmids as described in the figure legends. For detection of endogenous MDA5 binding to dsRNA, LYCK cells were treated with recombinant *Lc*IFNi (r*Lc*IFNi, 250 ng/ml) for 24 h to enhance endogenous MDA5 expression. Then, cells were lysed in lysis buffer (20 mM Tris‐HCl, pH 7.4, 150 mM NaCl, 1 mM EDTA, 40 U/mL RNase inhibitor, 8 mM DTT, and 1% NP‐40). Ten percent of the cell lysate was collected as the input sample, and 100 ng of biotinylated RNA was added to the remaining lysate. For competition assays, 100 ng of unlabeled RNA was included. The mixtures were incubated at 4°C for 1 h, followed by incubation with Pierce Streptavidin Agarose Resin (Thermo Scientific, cat#20347) at 4°C for an additional 2 h. Resin‐bound RNA‐protein complexes were eluted by boiling in SDS‐PAGE sample buffer.

### 4.8 Co‐IP and Western Blot

For tagged protein co‐IP assays, HEK293T or LYCK cells were seeded in 10 cm dishes (5 × 10^6^ cells/dish) and transfected with 5 µg of the indicated plasmids for 24 h. For endogenous protein complex analysis, LYCK cells were treated with r*Lc*IFNi (250 ng/ml) for 24 h to increase the endogenous expression level of MDA5. Cell lysates were prepared using an IP lysis buffer (50 mM Tris‐HCl, pH 7.4, 150 mM NaCl, 1 mM EDTA, 1% Triton X‐100, and 1 mM PMSF). For tagged protein IP, the supernatants were incubated overnight at 4°C with anti‐Flag, anti‐HA, or anti‐Myc magnetic beads (Beyotime; Cat#P2115, P2121, and P2118). Beads were washed three times with TBST, and bound proteins were eluted with the corresponding Flag, HA, or Myc peptides (Beyotime; Cat# P9801, P9808, P9805). Eluted proteins were resolved by SDS‐PAGE and transferred onto 0.2 µm PVDF membranes (Millipore, cat#ISEQ00010) using the Mini Trans‐Blot Cell system (Bio‐Rad). Membranes were blocked with TBST containing 5% nonfat dry milk for 1 h at room temperature. After overnight incubation with primary antibodies at 4°C, membranes were washed three times with TBST and incubated with HRP‐conjugated secondary antibodies (anti‐mouse or anti‐rabbit, as appropriate) for 1 h at room temperature. Signals were detected using an ECL chemiluminescent system. For endogenous protein IP, LYCK cells were treated with recombinant *Lc*IFNi (r*Lc*IFNi, 250 ng/ml) for 24 h to enhance endogenous MDA5 expression. The clarified lysates were incubated overnight at 4°C with an anti‐MDA5 antibody, followed by incubation with Protein A/G beads (Beyotime, Cat#P2108). After extensive washing, the immunoprecipitated proteins were subjected to Western blot analysis using the BeyoWB Clean‐Blot IP Detection Kit (Beyotime, Cat#P1830S) to minimize interference from the heavy and light chains of the immunoprecipitating antibody.

To assess the phosphorylation status of *Lc*IRF3, co‐IP was performed using a pan–phospho–serine/threonine antibody covalently crosslinked to Protein A/G beads (Beyotime, Cat#P2108) via DSS. This strategy previously applied to study IRF3 phosphorylation in teleosts [17, 59]. Briefly, LYCK cells were seeded in 10‐cm dishes (5×10^6^ cells/dish) were transfected with the indicated siRNAs for 24 h, followed by transfection with either 5′ppp‐dsRNA (2.5 µg/mL) or poly(I:C)‐HMW (2.5 µg/mL) for an additional 24 h. Cells were lysed in IP lysis buffer supplemented with protease and phosphatase inhibitor cocktails (Beyotime, Cat#P1045) for 30 min on ice. After centrifugation, clarified lysates were incubated overnight at 4°C with the crosslinked antibody–bead complex. The immunoprecipitated proteins were eluted by boiling in 2× SDS loading buffer and subjected to Western blot analysis to detect phosphorylated *Lc*IRF3.

### 4.9 AlphaFold3 Modeling

Structural models of *Lc*MDA5 in complex with either 5′ppp‐RNA duplex or 15‐mer blunt‐ended dsRNA were generated using AlphaFold3 [60]. To focus on the RNA‐binding and helicase regions, the input sequence for *Lc*MDA5 excluded the two N‐terminal CARDs (deleted residues 1–294). For the *Lc*MDA5 bound to a 5′ppp‐dsRNA, the RNA sequence was derived from the experimentally resolved structure of human RIG‐I in complex with a 5′ppp‐RNA duplex (PDB: 4AY2). The 5′ppp moiety was explicitly included as a ligand in the AlphaFold3 input. For the *Lc*MDA5‐blunt‐ended dsRNA complex, the RNA sequence was taken from the structure of mouse MDA5 bound to a 15‐mer blunt‐ended dsRNA (PDB: 7BKQ). In both models, Zn^2^^+^ and ADP were incorporated as cofactors to support correct folding and enhance structural stability. The monomeric *Lc*MDA5 model was also generated by AlphaFold3 in the absence of ligand. The interfaces were analyzed using the PDBePISA server [61].

### 4.10 MD Simulation

The AlphaFold3‐predicted structure of *Lc*MDA5 in complex with 15‐mer blunt‐ended dsRNA, as well as the predicted apo structure of *Lc*MDA5, were employed as initial models for MD simulations. Since the 5′ppp moiety was not covalently attached to the dsRNA terminus in the AlphaFold3 model, the 5′ppp‐RNA duplex in the *Lc*MDA5–5′ppp‐RNA complex was manually replaced with a crystallographically resolved 5′ppp‐RNA duplex structure (PDB ID: 4AY2) for subsequent MD simulations. The resulting complex was energy‐minimized using the Molecular Operating Environment (MOE) software [62]. All‐atom MD simulations were conducted using GROMACS version 2022.3 with the Amber99SB‐ILDN force field [63, 64]. Proteins were defined as receptors and RNA molecules as ligands. Each protein–RNA system was solvated in a cubic box of TIP3P water molecules, ensuring a minimum distance of 1.2 nm between the solute and the box boundary. Na^+^ and Cl^−^ ions were added to neutralize the system. Energy minimization was conducted using the steepest descent algorithm. Each system was equilibrated using both the canonical (NVT) and isothermal–isobaric (NPT) ensembles with 100,000 steps, a coupling constant of 0.1 ps, and a total duration of 100 ps. Temperature was maintained at 300 K using the velocity‐rescale (V‐rescale) thermostat. Pressure was controlled at 1 bar using the Berendsen barostat during equilibration. Production MD simulations were executed under NPT conditions for 200 ns with a 2 fs integration time step. Trajectory snapshots were recorded every 10 ps for subsequent analysis.

### 4.11 Normal Mode Analysis

NMA was performed using the iMODS server (https://imods.iqfr.csic.es/) to evaluate the motions and conformational deformability of the modeled *Lc*MDA5 structure [65]. The input models included the AlphaFold3‐predicted *Lc*MDA5–5′ppp‐RNA duplex complex and the corresponding *Lc*MDA5 model generated by removing the 5′ppp‐RNA duplex from the complex. All calculations were carried out using the default parameters provided by the server.

### 4.12 Immunofluorescence Assay

HEK293T cells or LYCK cells were cultured on the coverslips in a 6‐well culture plate (1×10^6^ cells/well) overnight, followed by transfection with plasmids (2 µg each) as indicated in the figures and figure legends. After 24 h, cells were washed three times with PBS and then fixed with 4% paraformaldehyde at room temperature for 15 min. Cells were permeabilized with 0.1% Triton X‐100 in PBS for 10 min, then blocked with 10% (v/v) goat serum for 60 min at room temperature, and incubated with primary antibody overnight at 4°C. After four 15‐min washes with PBS containing 1% goat serum, cells were incubated with CoraLite 594‐conjugated goat anti‐mouse IgG and CoraLite 488‐conjugated goat anti‐rabbit IgG for 2 h in a moist, dark environment at room temperature. Nuclei were labeled with 4',6‐diamidino‐2‐phenylindole (DAPI, 1 µg/mL), and the cells were imaged using a laser scanning confocal microscope (Leica TCS SP8).

### 4.13 Database Mining

To investigate the presence of RIG‐I gene remnants in the large yellow croaker, gene prediction analysis was conducted on the corresponding genomic region (GenBank accession: GCA_000972845.2; chromosome XIV: 6176481–13709732) using the FGENESH gene prediction tool [66]. The RIG‐I protein sequence from *D. rerio* was employed as a query in a tBLASTn search against the *L. crocea* genome to identify homologous fragments. The genomic environments surrounding RIG‐I or RNF135 in various species were analyzed using the Ensembl Genome Browser (release 114) and the NCBI database.

### 4.14 Statistical Analysis

Statistical analyses were performed using IBM SPSS Statistics (version 17.0). Comparisons between two groups were performed using two‐tailed unpaired Student's *t*‐tests, whereas comparisons among three or more groups were performed using one‐way analysis of variance (ANOVA) followed by Bonferroni post hoc tests. A *p*‐value < 0.05 was considered statistically significant. All bar charts show the mean ± SD of three independent biological replicates.

## Author Contributions

**Wenxing Li**: Conceptualization, methodology, investigation, writing – original draft. **Yuan Feng**: Investigation. **Huazhi Chen**: Visualization, investigation, data curation. **Jiamei Liu**: Methodology. **Álvaro Fernández Montero** and **Jiangtao Gao**: Writing – review & editing. **Jingqun Ao**: Writing – original draft. **Xinhua Chen**: Supervision, writing – review & editing.

## Conflicts of Interest

The authors declare no conflicts of interest.

## Supporting information




**Supporting File**: advs78318‐sup‐0001‐SuppMat.docx.

## Data Availability

The data that support the findings of this study are available from the corresponding author upon reasonable request.
