# Supplementary material for: A Fish MDA5 Integrates Canonical RIG‐I and MDA5 Ligand Recognition
Source: Adv Sci (Weinh). 2026 Oct 14:e78318. Online ahead of print. doi: 10.1002/advs.78318 (PMC13669214; doi:10.1002/advs.78318)
Supplement: Supplementary file 1 [file ADVS-9999-e78318-s001.docx]

Figure. S1

Figure. S1 5′ppp-dsRNA triggers type I IFN signaling through its 5′ppp moiety. LYCK cells seeded in 6-well plates (1 × 10⁶ cells/well) were either mock-transfected or transfected with 2.5 μg/mL 5′ppp-dsRNA or its negative control (lacking the 5′ppp moiety). At 24 h post-transfection, cells were collected for RT-qPCR analysis of type I IFNs. Data represent the mean ± SD from three independent experiments (**p* < 0.05, two-tailed unpaired Student’s *t*-test).

Figure. S2

Figure. S2 Protein flexibility analysis of *Lc*MDA5. (**A**) RMSF analysis of protein residues over 200-ns MD simulations, comparing the apo and dsRNA-bound states of *Lc*MDA5. RMSF values represent the positional flexibility of each residue throughout the simulations. The RNA-capping loop is highlighted in gray. (**B**) Protein deformability profile. The direction of motion is displayed with arrows. Arrows indicate the direction of motion, and the color spectrum from blue to red represents per-residue flexibility (blue, rigid; red, flexible). The figure indicates that ligand removal markedly increased overall protein flexibility. In the RNA-bound state, the RNA-capping loop showed constrained motion, and the protein-RNA complex behaved largely as a rigid unit. In contrast, removing the RNA made the RNA-capping loop more flexible and caused it to collapse downward, indicating that the "capping" state was lost.

Figure. S3

Figure. S3 Antiviral activity of *Lc*MDA5 against VSV infection. (A) Validation of VSV infection in LYCK cells. Following infection with VSV (MOI = 0.01), the expression of the viral G gene was examined at the mRNA and protein levels to confirm successful viral infection. (B) SDD-AGE analysis of endogenous *Lc*MDA5 oligomerization. LYCK cells were cultured in 6-well plates at 1 × 10⁶ cells/well and transfected with VSV-RNA or poly(I:C)-HMW at 2.5 μg/mL each. CIAP-treated VSV RNA, which lacks the 5′ppp moiety, was included as a control to assess the role of RNA 5′-terminal phosphorylation in *Lc*MDA5 activation. (C) Contribution of the *Lc*MDA5 5′ppp-binding pocket to antiviral defense against VSV. *Lc*MDA5-knockdown LYCK cells (6-well plates, 1 × 10⁶ cells/well) were reconstituted with an empty vector, WT *Lc*MDA5, or a 5′ppp-dsRNA recognition-defective *Lc*MDA5 mutant (2 μg), and infected with VSV (MOI = 0.01) for 12 h. Viral titers in culture supernatants were measured by TCID50 assays. Immunoblotting confirmed comparable expression levels of the transfected plasmids in LYCK cells. (D) *Lc*MDA5 mutants defective in 5′ppp-dsRNA recognition showed reduced induction of type I IFNs compared with WT *Lc*MDA5. *Lc*MDA5-knockdown LYCK cells were reconstituted with an empty vector, WT *Lc*MDA5, or 5′ppp-dsRNA recognition-defective *Lc*MDA5 mutants, followed by infection with VSV (MOI = 0.01) for 12 h. The mRNA levels of *Lc*IFNh, *Lc*IFNi, and *Lc*Viperin were quantified by RT-qPCR. Data are mean ± SD from three independent experiments (**p* < 0.05, one-way ANOVA followed by Bonferroni post hoc tests).

Figure. S4

Figure. S4 Protein-level validation of *Lc*STING and *Lc*MAVS knockdown. (A) LYCK cells were transfected with 100 nM siSTING#1 or siNC. (B) LYCK cells seeded in 6-well plates (1 × 10⁶ cells/well) were transfected with siMAVS#1 or siNC at a final concentration of 100 nM. At 48 h post-transfection, endogenous *Lc*STING and *Lc*MAVS were analyzed by immunoblotting using the corresponding antibodies. β-Tubulin served as a loading control.

Figure. S5

Figure. S5 Interaction of *Lc*MDA5 with *Lc*MAVS and *Lc*STING in LYCK cells. (**A**) *Lc*MAVS enhances the interaction between *Lc*MDA5 and *Lc*STING. LYCK cells cultured in a 10-cm dish were transfected or co-transfected with the indicated plasmids (5 μg each). Twenty-four hours post-transfection, protein-protein interactions were analyzed by Co-IP. (B) The CARDs of *Lc*MDA5 are essential for its interaction with both *Lc*MAVS and *Lc*STING. LYCK cells were transfected or co-transfected with the indicated plasmids (5 μg each). Twenty-four hours post-transfection, protein-protein interactions were analyzed by Co-IP.

Figure. S6

Figure. S6 Zebrafish MDA5 has the capacity to bind 5′ppp-dsRNA and poly(I:C)-HMW. (**A-B**) RNA pull-down assays showing binding of *Dr*MDA5 to biotinylated 5′ppp-dsRNA (A) or poly(I:C)-HMW (B). HEK293T cells were transfected with a plasmid encoding Flag-tagged *Dr*MDA5, and cell lysates were incubated with biotinylated RNA probes. Binding specificity was assessed using non-biotinylated competitors.

Figure. S7

Figure. S7 Structural comparison of predicted 5′ppp-dsRNA-bound MDA5 models from mouse and fish. In the predicted mouse MDA5–5′ppp-dsRNA complex, MDA5 adopts an open conformation similar to the previously reported mouse MDA5–dsRNA structure (as shown in Figure 4 C; PDB: 7BKQ), consistent with the canonical MDA5 dsRNA-binding mode rather than an RNA-end capping configuration. Predicted zebrafish and other fish MDA5–5′ppp-dsRNA complexes show a similar RNA-end capping configuration.

Table S1 Oligonucleotides in this study.

| Primer | Sequence (5′ to 3′ ) | Application |
| --- | --- | --- |
| LcMDA5-Flag-F | TGGAGCTGCAGTTGCTAAGCTTATGGCATCTGATAGCGACGAGT | Plasmid construction |
| LcMDA5-Flag-R | TTTGTAGTCAGCCCGGGATCCGTAAGTTTCTTCCTCATCTG |  |
| LcMDA5-ΔCARD-F | TGGAGCTGCAGTTGCTAAGCTTATGGATTATCAGACGGACGTTGC |  |
| LcMAVS-HA-F | CGGGCCCAGGCCCGAATTCATGTCGTTTGCCAGAGA |  |
| LcMAVS-HA-R | TCGTATGGGTAGCCGGTACCGTTCTTAAACTTCCACGCC |  |
| LcSTING-Flag-F | TGGAGCTGCAGTTGCTAAGCTTATGCTGTGCCTCCAGGATCAG |  |
| LcSTING-Flag-R | TTTGTAGTCAGCCCGGGATCCCATGAATCCCTGATAATT |  |
| LcSTING-myc-F | TGCTGGATATCTGCAGAATTCGCCACCATGCTGTGCCTCCAGGATCAG |  |
| LcSTING-myc-R | GTTCGGGCCCAAGCTTGGTACCCATGAATCCCTGATAATT |  |
| DrMDA5-Flag-F | TGGAGCTGCAGTTGCTAAGCTTATGGATCCAAACATGAGCAGC |  |
| DrMDA5-Flag-R | TTTGTAGTCAGCCCGGGATCCGTTAGTGTCCATATCTTCAT |  |
| LcIFNi-pGL3-F | CGGGTACCGAAATAAAAATCTTACTAATGAACAGTTG |  |
| LcIFNi-pGL3-R | CCCTCGAGTCCTCAGCTGGTGGTTGTG |  |
| siNC-sense | UUCUCCGAACGUGUCACGUTT | siRNA |
| siNC-antisense | ACGUGACACGUUCGGAGAATT |  |
| siMDA5#1-sense | CGGACUACAUGCAGCUGAA |  |
| siMDA5#1-antisense | UUCAGCUGCAUGUAGUCCG |  |
| siMDA5#2-sense | GGUGAUGUAAUCAAGACAA |  |
| siMDA5#2-antisense | UUGUCUUGAUUACAUCACC |  |
| siTLR3#1-sense | GCUACAGAAUACAUCUCUA |  |
| siTLR3#1-antisense | UAGAGAUGUAUUCUGUAGC |  |
| siTLR3#2-sense | GAUGUCAGUUACAACAGUA |  |
| siTLR3#2-antisense | UACUGUUGUAACUGACAUC |  |
| siTLR7#1-sense | CAAUCAGCUCGACAGUAUA |  |
| siTLR7#1-antisense | UAUACUGUCGAGCUGAUUG |  |
| siTLR7#2-sense | GGAUUGACUCACAUGCUUA |  |
| siTLR7#2-antisense | UAAGCAUGUGAGUCAAUCC |  |
| siMAVS#1-sense | CGACUCAGAUGAACUCAGA |  |
| siMAVS#1-antisense | UCUGAGUUCAUCUGAGUCG |  |
| siMAVS#2-sense | GGCAGAUGAGAUCAGAGCA |  |
| siMAVS#2-antisense | UGCUCUGAUCUCAUCUGCC |  |
| siMAVS#3-sense | GGAUGAACGUGGUGAUGAU |  |
| siMAVS#3-antisense | AUCAUCACCACGUUCAUCC |  |
| siSTING#1-sense | GACGACAGCAUCCAUUUCUAUG |  |
| siSTING#1-antisense | CAUAGAAAUGGAUGCUGUCGUC |  |
| siSTING#2-sense | GCACAGCGUCUACAGAGUA |  |
| siSTING#2-antisense | UACUCUGUAGACGCUGUGC |  |
| siSTING#3-sense | CGGUGGAGAACACCGACAA |  |
| siSTING#3-antisense | UUGUCGGUGUUCUCCACCG |  |
| LcIFNi-qF | ACAGCCAATCATCCTCAGTGCCA | RT-qPCR |
| LcIFNi-qR | AGCCATCCAGCCACAGGCAGCAC |  |
| LcIFNd-qF | CTTGTTTGTGTGCCTGTCTCTC |  |
| LcIFNd-qR | GTCCTCCACCTGAGCATCC |  |
| LcIFNh-qF | GGACTGGACAGGAGTGTCGAG |  |
| LcIFNh-qR | CAGGAAGCAGAGGTGAGGTTG |  |
| LcTNF-α1-qF | AACGCCTCACACCTCTCA |  |
| LcTNF-α1-qR | GTCCACAGTTTGTCTCCTCTATTC |  |
| LcIL-6-qF | CGACACACCCACTATTTACAAC |  |
| LcIL-6-qR | TCCCATTTTCTGAACTGCCTCT |  |
| LcIFN-γ-qF | TGAACACCTCGAGGCAAAGA |  |
| LcIFN-γ-qR | CTCCTTCCTGACCTCTCCGT |  |
| LcPKR-qF | GCACAGAAGATAGGCTCGGT |  |
| LcPKR-qR | TGTCTGACGAACTGGTGTGG |  |
| LcMxA-qF | GATGCTATAAGCCTCACCACA |  |
| LcMxA-qR | GTTGATAAATCCTGGCAGTTC |  |
| LcViperin-qF | TTCAGGCATGGAAAAGAT |  |
| LcViperin-qR | GACGATGCTGACACTTGG |  |
| Lcβ-actin-qF | GACCTGACAGACTACCTCATG |  |
| Lcβ-actin-qR | AGTTGAAGGTGGTCTCGTGGA |  |
| RIG-I-degenerate-F1 | ATHGTNGGNTTRACNGCN | Fish RIG-I amplification |
| RIG-I-degenerate-R1 | TCNGGNGTRTGNACRTA |  |
| RIG-I-degenerate-F2 | ATHGTNGGNYTNACNGCN |  |
| RIG-I-degenerate-R2 | TCYGGYGTRTGYACRTA |  |
